# Tensile Stress‐Activated and Exosome‐Transferred YAP/TAZ‐Notch Circuit Specifies Type H Endothelial Cell for Segmental Bone Regeneration

**DOI:** 10.1002/advs.202309133

**Published:** 2024-01-15

**Authors:** Feng Wang, Shanyu Li, Lingchi Kong, Kai Feng, Rongtai Zuo, Hanzhe Zhang, Yifan Yu, Kunqi Zhang, Yuting Cao, Yimin Chai, Qinglin Kang, Jia Xu

**Affiliations:** ^1^ Department of Orthopedics Shanghai Sixth People's Hospital Affiliated to Shanghai Jiao Tong University School of Medicine Shanghai 200233 China

**Keywords:** bone formation, distraction osteogenesis, endothelial cell, exosome, mechanical force

## Abstract

The Ilizarov technique has been continuously innovated to utilize tensile stress (TS) for inducing a bone development‐like regenerative process, aiming to achieve skeletal elongation and reconstruction. However, it remains uncertain whether this distraction osteogenesis (DO) process induced by TS involves the pivotal coupling of angiogenesis and osteogenesis mediated by type H endothelial cells (THECs). In this study, it is demonstrated that the Ilizarov technique induces the formation of a metaphysis‐like architecture composed of THECs, leading to segmental bone regeneration during the DO process. Mechanistically, cell‐matrix interactions‐mediated activation of yes‐associated protein (YAP)/transcriptional co‐activator with PDZ‐binding motif (TAZ) transcriptionally upregulates the expression of Notch1 and Delta‐like ligand 4, which act as direct positive regulators of THECs phenotype, in bone marrow endothelial cells (BMECs) upon TS stimulation. Simultaneously, the Notch intracellular domain enhances YAP/TAZ activity by transcriptionally upregulating YAP expression and stabilizing TAZ protein, thus establishing the YAP/TAZ‐Notch circuit. Additionally, TS‐stimulated BMECs secrete exosomes enriched with vital molecules in this positive feedback pathway, which can be utilized to promote segmental bone defect healing, mimicking the therapeutic effects of Ilizarov technique. The findings advance the understanding of TS‐induced segmental bone regeneration and establish the foundation for innovative biological therapeutic strategies aimed at activating THECs.

## Graphical abstract

Tensile stress‐induced activation of yes‐associated protein/transcriptional co‐activator with PDZ‐binding motif‐Notch positive feedback loop specifies type H endothelial cells (THEC) during distraction osteogenesis. Vital molecules involved in this circuit are enriched in exosomes for intercellular communication of regenerative signals, which can be further utilized to promote segmental bone regeneration through THEC activation, mimicking the therapeutic effects of the Ilizarov technique.

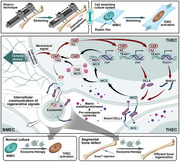

## 1 Introduction

From the earliest stages of embryonic development to diseases manifesting later in life, mechanical forces play a pivotal role across various biological levels, spanning from macroscopic biomechanics to microscopic mechanobiology.^[^ ^1^ ^]^ The skeletal system serves as the body's mechanical framework, constantly encountering a dynamic external mechanical environment, with bone homeostasis intricately regulated by mechanical forces.^[^ ^2^ ^]^ Dating back to Galileo's discovery of the relationship between loading and bone morphology in 1638, over two centuries of accumulated research in bone biomechanics led to the formulation of Wolff's Law in 1892, explicitly delineating the positive regulatory effects of mechanical loads on bone formation.^[^ ^3^ ^]^ Driven by this understanding, orthopedic surgery rapidly evolved, establishing critical clinical concepts such as appropriate exercise for osteoporotic patients, early postoperative weight‐bearing for fracture patients, and avoiding stress shielding in device fixation, all aimed at enhancing bone mass or promoting bone healing.^[^ ^4^ ^]^ However, the challenge of adequately promoting bone regeneration through mechanical loading remains, especially in cases of trauma, tumor resection, infection, and skeletal abnormalities, where the reconstruction of skeletal continuity proves to be a persistent clinical dilemma.^[^ ^5^ ^]^

Excitingly, since Ilizarov proposed the “tension‐stress principle” in 1989, the discovery of the unique stimulatory effects of tensile stress (TS) on bone regeneration has opened a new chapter in utilizing mechanical forces to regulate bone formation.^[^ ^6^ ^]^ The Ilizarov technique, employed to induce distraction osteogenesis (DO), is now a widely utilized orthopedic surgical approach that offers therapeutic advantages in overcoming the challenges of segmental bone regeneration in cases of limb lengthening and reconstruction.^[^ ^7^ ^]^ However, despite continuous refinements in the Ilizarov technique within clinical practices of orthopedic and plastic surgery, the slow mineralization rate of newly formed bone in the distraction zone remains a major challenge faced by both medical practitioners and patients.^[^ ^8^ ^]^ To address this issue, one approach involves exploring physicochemical or biological auxiliary measures directly based on the DO model, which is currently the predominant approach adopted in most relevant studies.^[^ ^9^ ^]^ Additionally, recognizing the unique biological characteristics of the DO model distinct from mere segmental bone defect (SBD) healing, and subsequently improving or transforming the Ilizarov technique, represents another highly promising strategy for limb lengthening and reconstruction research.^[^ ^10^ ^]^ Nonetheless, further exploration of the molecular mechanisms in DO process is imperative. TS acts on cells through cell‐matrix interactions, and subsequently, cells perceive and respond to mechanical signals by integrating and activating various pathways.^[^ ^11^ ^]^ In this mechano‐biological transduction process, yes‐associated protein (YAP)/transcriptional co‐activator with PDZ‐binding motif (TAZ) has been identified as a central mediator, primarily reported to enhance the osteogenic differentiation of mesenchymal stem cells in the distraction zone.^[^ ^12^ ^]^ Although numerous other crucial factors affecting bone regeneration, such as angiogenesis, inflammation regulation, and stem cell recruitment, have been confirmed to participate in the DO process,^[^ ^13^ ^]^ the mechanistic relationships with the mechano‐biological transduction downstream of TS, represented by YAP/TAZ, are still awaiting further exploration.

Characterized by robust expression of CD31 and endomucin (EMCN), type H endothelial cells (THECs) in bone have gained recognition for their pivotal role in coupling angiogenesis and osteogenesis during skeletal development.^[^ ^14^ ^]^ Specifically, THECs primarily distributed in the metaphysis play a crucial role in the recruitment and activation of osterix (OSX)‐positive osteoprogenitors through paracrine pathways, including the secretion of platelet‐derived growth factor (PDGF), transforming growth factor (TGF), and fibroblast growth factor (FGF).^[^ ^14^ ^]^ Moreover, their secretion of matrix metalloproteinases (MMP), especially MMP9, is instrumental in digesting the cartilage matrix of the growth plate, thereby significantly contributing to the leading structures during endochondral ossification for epiphyseal bone growth.^[^ ^15^ ^]^ Furthermore, the in vivo abundance of THECs and associated osteoprogenitors rapidly declines with age, which has been confirmed to be associated with age‐related bone loss.^[^ ^16^ ^]^ While the concept of coupling angiogenesis and osteogenesis has recently gained attention in bone regeneration research, the abundance of THECs has primarily been considered a biomarker for assessing neoangiogenesis within the healing area.^[^ ^17^ ^]^ The physiological role of THECs during bone regeneration remains ambiguous, as their unique bioactivities have been largely overlooked. Moreover, in contrast to the metaphysis, which receives significant attention in studies related to skeletal development and bone mass maintenance, the diaphyseal region, the primary focus of bone regeneration research, is inherently deficient in the distribution of THECs.^[^ ^14^ ^]^ Therefore, it remains uncertain whether authentic activation of THECs‐mediated coupling of angiogenesis and osteogenesis can occur and how it can be artificially regulated within the healing callus to achieve regeneration of SBD.

Under physiological conditions, THECs have been confirmed to be influenced by various signals, including slit guidance ligand 3 (slit3) from osteoblasts, PDGF‐BB from preosteoclasts, and cell‐matrix signals and Notch activity from endothelial cells themselves.^[^ ^15^, ^18^ ^]^ Among these various upstream signals involved in the coupling of angiogenesis and osteogenesis, Notch1/Delta‐like ligand 4 (Dll4) signaling plays a crucial role in inducing the THECs phenotype.^[^ ^18b^ ^]^ This is distinct from the well‐established suppressive function of Notch and its ligand Dll4 on endothelial sprouting in other organs and tumors.^[^ ^19^ ^]^ Previous studies have shown that the abundance of THECs is highly dependent on inducible Notch activation, achievable through different approaches such as direct genetic modification, administration of recombinant Dll4 protein, post‐transcriptional regulation, and epigenetic regulation, exhibiting therapeutic potential in pathological conditions such as aging‐ and ovariectomy‐induced osteoporosis.^[^ ^16^, ^20^ ^]^ Furthermore, in comparison to type L endothelial cells (TLECs) with low expression of CD31 and EMCN, upregulated gene sets in THECs showed a striking enrichment of extracellular matrix, basement membrane, and cell adhesion components, indicating the activation of cell‐matrix signals.^[^ ^18a^ ^]^ Among these genes, the representative *Itgb1*, encoding integrin β1, has been confirmed to be associated with maintaining the normal morphological and functional properties of THECs, although it does not affect their abundance.^[^ ^18a^ ^]^ Additionally, slit3 secreted by osteoblasts can promote THECs’ endothelium formation in vivo by acting on bone marrow endothelial cells (BMECs) through the roundabout guidance receptor 1 (ROBO1), and YAP has been found to act downstream of the slit3/ROBO1 pathway to control endothelial tube formation in vitro.^[^ ^18c^ ^]^ While the backgrounds and objectives of these diverse studies are not directly related to mechanical signals, it is intriguing that key molecules identified in their research results are coincidentally involved in cellular regulatory processes induced by mechanical tension, such as cell‐matrix interactions and the transduction of mechano‐biological signals.^[^ ^21^ ^]^ However, whether the mechanical TS during the DO process can stimulate the endothelium formation of THECs remains uncertain. Investigating this question necessitates an exploration of the potential regulatory role of the core transcriptional co‐activators YAP/TAZ in the characteristic Notch signaling of THECs phenotype in BMECs.

The regulatory pattern of YAP/TAZ on Notch signaling, although not yet studied in bone development and regeneration, exhibits significant context‐dependence across various physiological processes.^[^ ^22^ ^]^ In the skin of newborn mice, YAP overexpression leads to a reduction in Notch transcriptional responses, meanwhile conditional YAP/TAZ knockout triggers Notch signaling, resulting in the differentiation of epidermal progenitors.^[^ ^23^ ^]^ Conversely, YAP/TAZ has also been reported as an upstream regulator capable of activating its downstream functionally important effector, Notch signaling, to control liver cell fate.^[^ ^24^ ^]^ This activation governs liver growth and represses tumor initiation, albeit involving Notch2 and Jagged1 instead of Notch1/Dll4, achieved through interaction with TEA domain transcription factor (TEAD) 4 and binding to both their promoters.^[^ ^24^ ^]^ Given that the liver is the only organ, besides bone, where THECs are found,^[^ ^14^ ^]^ this strongly encourages exploration to determine whether a comparable activation effect of YAP/TAZ on Notch1/Dll4 also exists in THECs within the skeletal system. Simultaneously, the overexpression of Notch intracellular domain (NICD) in liver cancer cells has been reported to reciprocally upregulate the protein levels of YAP/TAZ,^[^ ^24a^ ^]^ providing a reference for the regulatory modes of both in THECs. Validating these hypotheses based on the DO model would not only enhance the understanding of the mechanisms underlying Ilizarov technique‐induced segmental bone regeneration but could also potentially contribute to translating the molecular pathways inducing THECs during the DO process into therapeutic approaches, offering a novel direction for limb lengthening and reconstruction.

In this study, we illustrate the enrichment of cell‐matrix signals and activation of YAP/TAZ in the regenerative tissue during the DO process, resulting in the activation of Notch1/Dll4 signaling and an increased abundance of THECs within the distraction area. These THECs play a pivotal role in establishing a metaphysis‐like architecture on both sides, thereby promoting directed bone regeneration. Mechanistically, in BMECs stimulated by TS, the activated YAP/TAZ upregulates the expression levels of Notch1 and Dll4 at the transcriptional level by interacting with TEAD1 and binding to their respective promoters, leading to the phenotypic transformation into THECs. Concurrently, NICD also upregulates YAP transcription and inhibits TAZ ubiquitination and degradation, forming a positive feedback regulatory network to assist in the activation of regenerative signals. Furthermore, exosomes derived from TS‐stimulated BMECs carry essential molecules involved in this YAP/TAZ‐Notch circuit, potentially playing a role in transmitting and maintaining regenerative signals initiated by TS in the bone regeneration area. These findings have significant implications for the development of a biological treatment for SBD, inspired by Ilizarov technique, by leveraging the therapeutic potential of these exosomes to mimic the regenerative effects of TS stimulation. In summary, our results provide a solid theoretical foundation for the Ilizarov technique and establish a promising therapeutic strategy to enhance the THECs‐mediated coupling of angiogenesis and osteogenesis during bone regeneration.

## 2 Results

### 2.1 Mechano‐Biological Transduction‐Dependent THECs Form a Metaphysis‐like Architecture During DO

**Figure 1:**
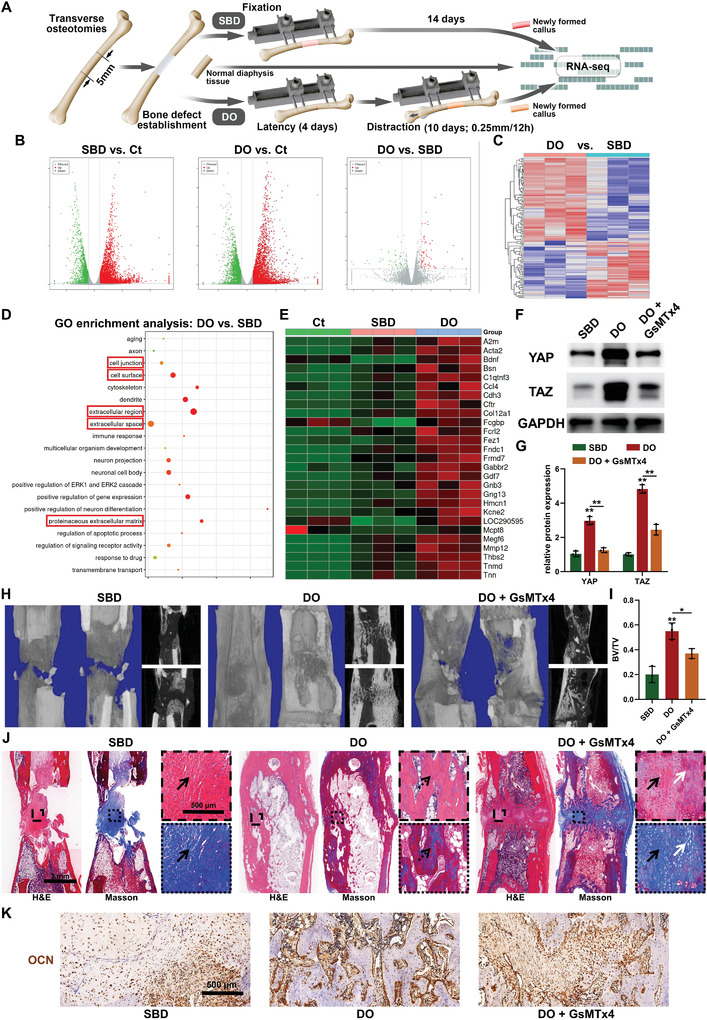
Mechanical signals‐dependent segmental bone regeneration during distraction osteogenesis (DO). A) Schematic representation of transcriptomic sequencing comparing the segmental bone defect (SBD) model and the DO model, with normal tibial tissue serving as a control (Ct). B) Volcano plots of RNA‐seq analysis among tissues from the midshaft of the normal tibia, healing callus of SBD, and regenerated tissue during DO at two weeks post‐surgery. C) Heatmap of RNA‐seq analysis between healing callus of SBD and regenerated tissue during DO) D) GO enrichment analysis of upregulated genes in the healing callus during DO process compared to the regenerated tissue during SBD healing indicating activated cell‐matrix signals (red rectangle). E) Heat map of differentially expressed genes involved in cell‐matrix signals in healing callus of SBD and DO groups compared to Ct group. F,G) Western blot images F) and quantitative analysis G) reveal the expression of YAP/TAZ signaling in the regenerated callus at three weeks post‐surgery. H,I) Images of three‐dimensional reconstruction (left) and coronal and sagittal sections (right) H) and bone volume/tissue volume (BV/TV) quantitative analysis I) based on the micro‐CT data collected at five weeks post‐surgery. J) H&E (left, top) and Masson (right, down) staining of the regenerated callus in different groups at five weeks post‐surgery. Black arrows: fibrous‐like tissue. White arrows: cartilaginous tissue. Dotted arrows: regenerated trabecular bone. K) Immunohistochemical staining of OCN in the healing area of different groups at five weeks post‐surgery. ^*^ *p <* 0.05, ^**^ *p <* 0.01.

To elucidate the physiological mechanisms governing segmental bone regeneration facilitated by the Ilizarov technique, comparable models of SBD and DO were established, and RNA‐seq analysis was conducted on the newly formed callus in both groups (**Figure** **1**A). The findings revealed substantial disparities in gene expression within the callus of the SBD and DO groups when compared to normal diaphysis tissue, with a deviation of 24.42% and 26.87% in gene expression, respectively (Figure 1B). Furthermore, over 90% of the differentially expressed genes were identified as repetitive across both groups (Figure 1B). Upon further comparison of the gene expression profiles between the SBD and DO groups, 117 differentially expressed genes were identified, constituting 0.52% of all detected genes (Figure 1B,C). Notably, the upregulated genes in the DO group exhibited significant enrichment in cell junctions, extracellular space, and proteinaceous extracellular matrix compared to the SBD group (Figure 1D). These genes generally exhibited slightly higher expression levels in the callus of the SBD group compared to normal diaphysis tissue but were markedly elevated in the DO group (Figure 1E), suggesting their potential association with activated segmental bone regeneration facilitated by the Ilizarov technique. In continuation of this investigation, the expression of YAP/TAZ signaling within the regenerative zones of the SBD and DO groups was evaluated, given their pivotal role as transcriptional co‐activators converting mechanical signals, transmitted through cell‐matrix interactions, into biological signals.^[^ ^12a^ ^]^ The results demonstrated the anticipated upregulation of YAP and TAZ expression in the DO group compared to the SBD group (Figure 1F,G). Moreover, the reduction of local mechanical sensitivity by the application of GsMTx4^[^ ^25^ ^]^ to the distraction zone resulted in diminished expression of YAP/TAZ (Figure 1F,G), further emphasizing the potential role of YAP/TAZ as key mediators of mechano‐biological transduction in the DO process. Subsequent micro‐CT analysis unveiled a significantly higher mineralization rate in the regenerative zone of the DO group compared to the SBD group, evident in the continuity of the cortical bone and increased BV/TV five weeks post‐surgery (Figure 1H,I). However, the application of GsMTx4 noticeably impeded the bone regeneration activated by the Ilizarov technique, leading to incomplete mineralization in the distraction zone and reduced BV/TV compared to the DO group (Figure 1H,I). Histological examination also revealed that, after five weeks post‐surgery, the regenerative area in the DO group featured well‐connected cortical bone primarily composed of newly formed bone trabeculae (Figure 1J). In contrast, both the SBD group and the DO + GsMTx4 group retained significant non‐mineralized tissues such as fibers and cartilage in the regenerative area (Figure 1J). Additionally, immunohistochemical results demonstrated a more pronounced expression of OCN in the regenerative tissue of the DO group compared to the SBD group and DO + GsMTx4 group (Figure 1K), indicating a locally heightened osteogenic activity. In conclusion, based on the comparable SBD and DO models established in this study, the molecular expression analysis and bone regeneration assessments predominantly underscore the significance of mechano‐biological transduction signaling downstream of TS, particularly indicating the pivotal role of YAP/TAZ in Ilizarov technique‐induced segmental bone regeneration.

**Figure 2:**
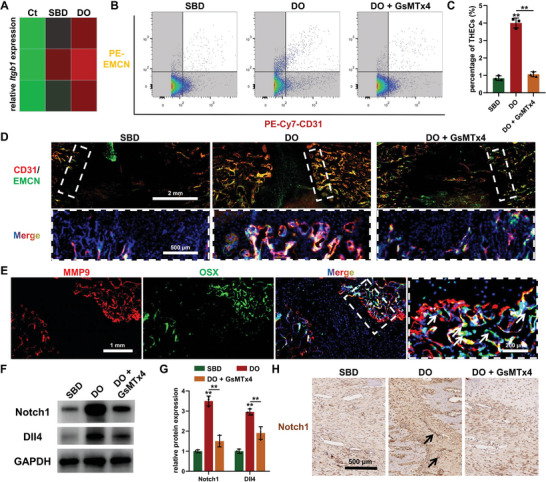
Mechanical signals‐dependent activation of type H endothelial cells (THECs) during distraction osteogenesis (DO). A) Differential gene expression of the representative cell‐matrix interaction molecule, *Itgb1*, in the segmental bone defect (SBD) and DO models. B,C) Flow cytometry B) and quantitative analysis C) of THECs abundance within bone healing area of SBD and DO (with or without GsMTx4 treatment) models at three weeks post‐surgery. D) Immunofluorescence staining of CD31 and EMCN for the detection of THECs distribution within bone healing areas in different groups at three weeks post‐surgery. E) Immunofluorescence staining of MMP9 and OSX for evaluating the potential osteogenesis‐coupling function of THECs in the distraction zone at three weeks post‐surgery. White arrows: OSX‐positive osteoprogenitors. F,G) Western blot images F) and quantitative analysis G) revealing the expression of Notch1/Dll4 signaling in the regenerated callus at three weeks post‐surgery. H) Immunohistochemical staining of Notch1 in the healing area of different groups at three weeks post‐surgery. Black arrows: Notch1‐positive vascular‐like structures. ^**^ *p <* 0.01.

THECs, characterized by elevated expression of CD31 and EMCN, are widely recognized as regulators of the coupling of angiogenesis and osteogenesis in bone development^[^ ^14^ ^]^; however, their precise physiological role in bone regeneration remains incompletely understood. Previous studies have suggested the enrichment of cell‐matrix signals in THECs, with *Itgb1* identified as a representative gene capable of regulating the structure and function of metaphyseal THECs, although the comprehensive gene expression profile has not been reported.^[^ ^18a^ ^]^ In the current study, transcriptome sequencing results from regenerative tissues in SBD and DO models not only confirmed the enrichment of cell‐matrix signals, as previously mentioned but also revealed higher expression levels of *Itgb1* in the DO group compared to the SBD group (**Figure** **2**A). To investigate whether and how THECs play a regulatory role in the DO process, flow cytometry was initially employed to assess the THECs content in regenerative tissues three weeks postoperatively. The results demonstrated a significantly higher proportion of THECs in the regenerative tissue during the DO process compared to the SBD model, with GsMTx4 intervention markedly reducing THECs proportions in the distraction zone (Figure 2B,C). Additionally, immunofluorescence staining revealed a scarcity of THECs in the regenerative region of SBD model (Figure 2D). In contrast, in the DO group, the formation of columnar tubes and arches consisting of THECs was observed on both sides of the distraction zone (Figure 2D). These structures closely resembled the arrangements of THECs near the epiphyseal growth plate, as reported in previous studies.^[^ ^14^ ^]^ Furthermore, in light of the documented functions of THECs in bone development, particularly in recruiting and activating OSX‐positive osteoprogenitors and secreting MMP9 to degrade the extracellular matrix,^[^ ^14^, ^15^ ^]^ MMP9 and OSX immunofluorescence double staining was employed to preliminarily assess the functionality of THECs in the distraction zone of the DO group. The results revealed that the metaphysis‐like structures developed by THECs during DO exhibited a significant expression of MMP9 and were selectively positioned around OSX‐positive osteoprogenitors (Figure 2E), mirroring the characteristics of THECs observed during bone development.^[^ ^14^, ^15^ ^]^ These findings suggest a potential role of THECs in activating the regenerative potential of bone tissue during DO by contributing to extracellular matrix resorption in the cartilaginous callus and the recruitment of osteoprogenitors. Furthermore, the application of GsMTx4 in the distraction zone significantly reduced the abundance of THECs in the DO process and disrupted the formation of columnar tubes and arches, as indicated by both flow cytometry and immunofluorescence results (Figure 2B–D). This outcome aligns with the inhibited YAP/TAZ signaling and impaired bone regeneration, indicating the potential involvement of THECs in regulating the DO process and their association with YAP/TAZ‐mediated mechano‐biological transduction. As a characteristic signal of THECs phenotype,^[^ ^18b^ ^]^ the expression of Notch1/Dll4 also exhibited a similar trend, with significantly higher levels in the regenerative zone of the DO group compared to the SBD group, notably inhibited by GsMTx4 intervention (Figure 2F,G). Immunohistochemical results similarly demonstrated a more pronounced Notch1 expression in the regenerative zone of the DO group, especially in Notch1‐positive vascular‐like structures, compared to the SBD group and DO + GsMTx4 group (Figure 2H).

**Figure 3:**
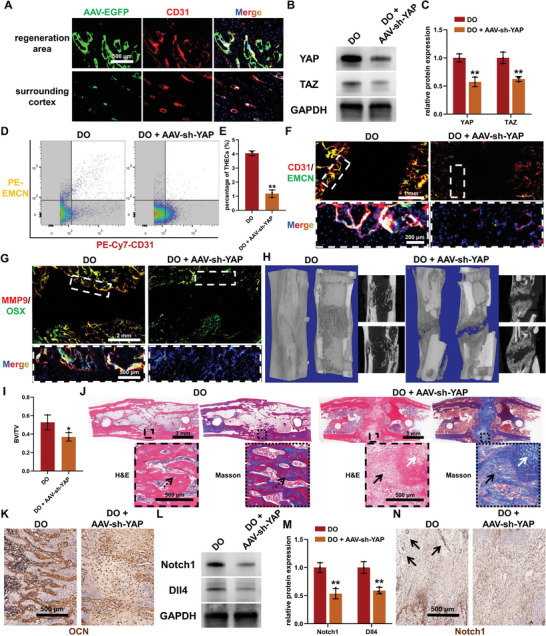
YAP/TAZ‐mediated mechano‐biological transduction for type H endothelial cells (THECs) activation and bone regeneration during distraction osteogenesis (DO). A) The co‐localization of EGFP, delivered by an endothelial cell‐specific adeno‐associated virus (AAV), with CD31‐positive endothelial cells in bone tissue substantiating the specificity of endothelial cell transfection. B,C) Western blot images B) and quantitative analysis C) revealing the downregulation of YAP/TAZ signaling in the regenerated callus of DO model with AAV‐mediated endothelial cell‐specific YAP knockdown (AAV‐sh‐YAP). D,E) Flow cytometry D) and quantitative analysis E) of THECs abundance within bone healing area of DO and DO + AAV‐sh‐YAP groups at three weeks post‐surgery. F) Immunofluorescence staining of CD31 and EMCN for the detection of THECs distribution within bone healing areas in different groups at three weeks post‐surgery. G) Immunofluorescence staining of MMP9 and OSX for evaluating the potential osteogenesis‐coupling function of THECs in the distraction zone in different groups at three weeks post‐surgery. H,I) Images of three‐dimensional reconstruction (left) and coronal and sagittal sections (right) H) and bone volume/tissue volume (BV/TV) quantitative analysis I) based on the micro‐CT data collected at five weeks post‐surgery. J) H&E (left) and Masson (right) staining of the regenerated callus at five weeks post‐surgery. Black arrows: fibrous‐like tissue. White arrows: cartilaginous tissue. Dotted arrows: regenerated trabecular bone. K) Immunohistochemical staining of OCN in the healing area at five weeks post‐surgery. L,M) Western blot images L) and quantitative analysis M) revealing the expression of Notch1/Dll4 signaling in the regenerated callus at three weeks post‐surgery. N) Immunohistochemical staining of Notch1 in the healing area of different groups at three weeks post‐surgery. Black arrows: Notch1‐positive vascular‐like structures. ^*^ *p <* 0.05, ^**^ *p <* 0.01.

To comprehensively elucidate the regulatory impact of YAP/TAZ‐mediated mechano‐biological transduction on THECs characterized by Notch1/Dll4 signaling, an AAV with the endothelial cell‐specific serotype ENT,^[^ ^26^ ^]^ carrying the endothelial cell‐specific promoter TIE,^[^ ^27^ ^]^ was employed to selectively knockdown YAP expression in BMECs within the regenerative region of the DO model. The vector's specificity for BMECs was initially validated through the detection of indicative EGFP and CD31 immunofluorescence staining, demonstrating distinct co‐localization of AAV‐carried EGFP with CD31‐positive BMECs in both the bone regeneration region and surrounding bone tissues (**Figure** **3**A). Simultaneously, the administration of AAV carrying sh‐YAP significantly reduced YAP expression levels in the regenerative tissue of the DO model (Figure 3B,C), collectively signifying the successful selective inhibition of mechano‐biological transduction signaling in the BMECs of the distraction zone. The observed reduction in TAZ expression (Figure 3B,C) may be attributed to the closely interrelated functions and shared regulatory networks between YAP and TAZ, aligning with previous reports.^[^ ^28^ ^]^ Results from flow cytometry analysis exhibited a substantial decrease in the proportion of THECs in the distraction zone three weeks postoperatively following the application of AAV‐sh‐YAP, and the metaphysis‐like architecture formed by THECs was visibly disrupted, as confirmed by immunofluorescence staining (Figure 3D–F). Moreover, MMP9 and OSX immunofluorescence double staining demonstrated a notable disruption in the expression and distribution of both markers due to the application of AAV‐sh‐YAP (Figure 3G), further corroborating the loss of function in THECs in the distraction zone, potentially contributing to inadequate mineralization in the DO process. As anticipated, micro‐CT imaging confirmed a significant reduction in the mineralization rate of DO processes upon the BMECs‐specific suppression of mechano‐biological transduction signaling, as evidenced by the discontinuous cortical bone and reduced BV/TV in the distraction zone five weeks postoperatively (Figure 3H,I). Similarly, histological examination results revealed that compared to the DO group, the regenerative region of the DO + AAV‐sh‐YAP group retained conspicuous non‐mineralized tissues, including fibrous and cartilaginous components, after five weeks, accompanied by a suppressed expression of OCN (Figure 3J,K). Additionally, three weeks postoperatively in the DO group, a notable expression of Notch1, especially Notch1‐positive vascular‐like structures, in the regenerative zone was observed, which could be significantly disrupted by the application of AAV‐sh‐YAP, accompanied by a decrease in the expression levels of Dll4 (Figure 3L–N). These findings are consistent with the impairments observed in the abundance, structure, and function of THECs in the DO + AAV‐sh‐YAP group. In summary, the promoting function in bone regeneration of THECs, characterized by Notch1/Dll4 signaling, during the DO process has been preliminarily established, with indications of potential regulation by YAP/TAZ‐mediated mechano‐biological transduction. This underscores the necessity for further investigations into the regulatory mechanisms of YAP/TAZ on Notch1/Dll4 signaling in BMECs under TS stimulation.

### 2.2 TS Stimulation Activates YAP/TAZ‐Notch Circuit and Specifies THECs

**Figure 4:**
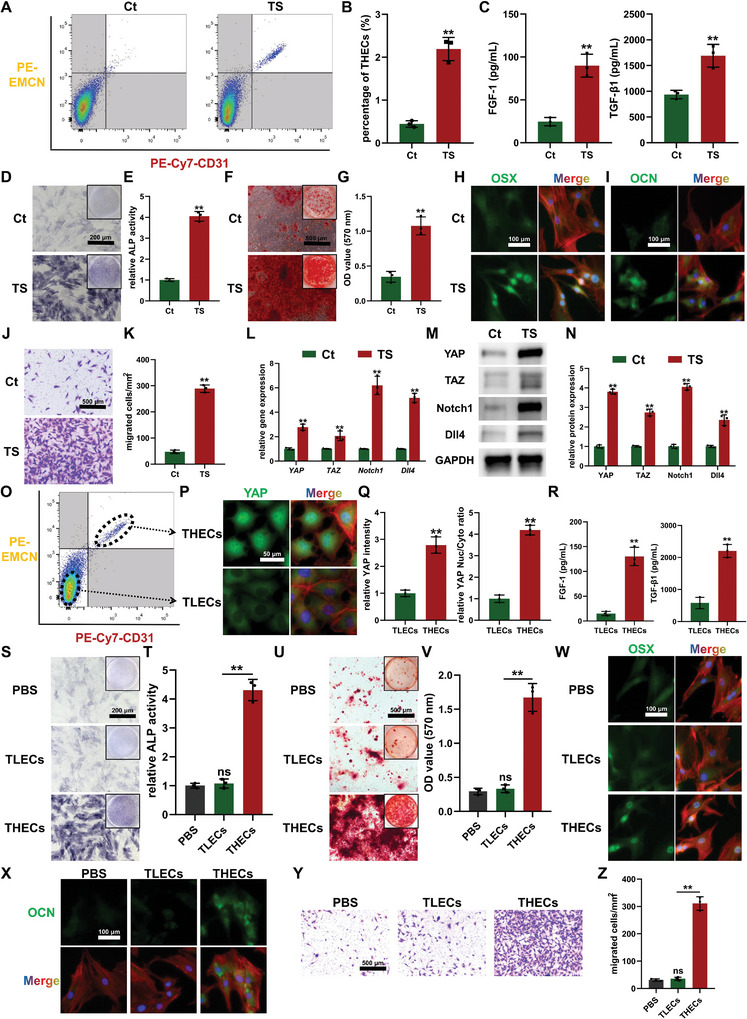
Tensile stress (TS)‐induced type H endothelial cells (THECs) phenotype and the involvement of YAP/TAZ signaling. A,B) Flow cytometry A) and quantitative analysis B) of THECs phenotype after mechanical TS stimulation on bone marrow endothelial cells (BMECs). C) Concentrations of FGF‐1 and TGF‐β1 in conditioned medium (CM) from TS‐stimulated BMECs. D,E) Alkaline phosphatase (ALP) staining D) and activity measurement E) of bone marrow mesenchymal stem cells (BMSCs) after seven days of osteogenic induction with CM treatment. F,G) Alizarin red S staining F) and quantitative analysis G) of calcium nodules after fourteen days of osteogenic induction with CM treatment. H,I) Immunofluorescence staining of OSX H) and OCN I) for the evaluation of osteogenic differentiation of BMSCs. J,K) Transwell assay images J) and quantitative analysis K) revealing the chemotaxis of BMSCs in the upper chambers towards BMECs in the lower chambers. L) The gene expression of Notch1/Dll4 and YAP/TAZ pathways in TS‐stimulated BMECs. M,N) Western blot images K) and quantitative analysis L) revealing the expression of Notch1/Dll4 and YAP/TAZ pathways in TS‐stimulated BMECs. O) Fluorescence‐activated cell sorting (FACS) for respectively isolating type L endothelial cells (TLECs) and THECs from TS‐stimulated BMECs. P,Q) Immunofluorescence staining P) and quantitative analysis Q) for evaluating the expression and nuclear translocation of YAP in FACS‐sorted TLECs and THECs. R) Concentrations of FGF‐1 and TGF‐β1 in CM from FACS‐sorted TLECs and THECs. S,T) ALP staining S) and activity measurement T) of BMSCs after osteogenic induction with CM treatment. U,V) Alizarin red S staining U) and quantitative analysis V) of calcium nodules of BMSCs in different groups. W,X) Immunofluorescence staining of OSX W) and OCN X) for the evaluation of osteogenic differentiation of BMSCs. Y,Z) Transwell assay images Y) and quantitative analysis Z) revealing the chemotaxis of BMSCs towards FACS‐sorted TLECs and THECs. ^ns^ *p >* 0.05, ^**^ *p <* 0.01.

To simulate the mechanical microenvironment of DO process in vitro, a cell stretching culture system was employed on BMECs. Flow cytometry analysis revealed a notable increase in the proportion of THECs in BMECs following the TS stimulation (**Figure** **4**A,B). Furthermore, ELISA results demonstrated that BMECs exposed to TS secreted higher levels of FGF‐1 and TGF‐β1 (Figure 4C), which aligns with previous studies highlighting the specific upregulation of *Fgf1* and *Tgfb1* genes in THECs.^[^ ^14^ ^]^ To elucidate the role of TS‐stimulated BMECs in coupling osteogenesis, BMSCs were treated with CM from BMECs and simultaneously induced osteogenic differentiation. The results demonstrated that, compared to the Ct group, CM from the TS group significantly augmented ALP activity in BMSCs during early osteogenic differentiation and facilitated pronounced calcium nodule formation in the later stages (Figure 4D–G). Simultaneously, immunofluorescence results revealed a substantial upregulation of the expression of the classical osteogenic markers, OSX and OCN,^[^ ^29^ ^]^ within BMSCs treated with CM from the TS group (Figure 4H,I). Moreover, as revealed by a Transwell co‐culture system of BMECs and BMSCs, TS‐stimulated BMECs in the lower chamber exerted superior chemotactic effects, leading to enhanced migration of BMSCs from the upper chamber to the lower chamber (Figure 4J,K). These collective findings confirm the augmentative impact of TS on the role of BMECs in recruiting BMSCs and activating their osteogenic differentiation through paracrine signaling. Additionally, a significant upregulation of YAP, TAZ, Notch1, and Dll4 in BMECs was noted following TS stimulation in both mRNA and protein expression levels (Figure 4L–N), consistent with the findings observed in the DO model. Concurrently, the canonical downstream target gene of the Notch pathway, *HES1*, demonstrated a noteworthy upregulation in expression within the TS group, offering additional confirmation of Notch signaling activation (Figure S1, Supporting Information).

To further elucidate whether the impact of the TS stimulation on the regulatory function of BMECs in osteogenesis is directly associated with the activation of THECs, TLECs, and THECs were respectively isolated from TS‐stimulated BMECs through FACS for further examination (Figure 4O). Immunofluorescence staining results revealed a distinct upregulation and nuclear translocation of YAP molecules in THECs compared to TLECs (Figure 4P,Q), consistent with previous in vivo and in vitro molecular expression findings. This further suggests the involvement of YAP/TAZ‐mediated mechano‐biological transduction in specifying THECs phenotype. Additionally, ELISA results demonstrated significantly higher levels of FGF‐1 and TGF‐β1 in the CM derived from THECs compared to TLECs (Figure 4R). On this basis, TLECs did not exhibit a promotive effect on the osteogenic differentiation and migration capacity of BMSCs, which was evident as ALP activity, calcium nodule formation, and the expression of osteogenic markers remained unchanged in response to CM derived from TLECs (Figure 4S–X), and the number of migrated BMSCs in the Transwell assay with TLECs co‐culture did not show a significant difference compared to the PBS group (Figure 4Y,Z). In contrast, CM derived from THECs significantly enhanced ALP activity, calcium nodule formation, and the expression of osteogenic markers such as OSX and OCN in BMSCs (Figure 4S–X), confirming a substantial enhancement in the osteogenic differentiation activity of BMSCs. Furthermore, the quantity of BMSCs, co‐cultured with THECs, that migrated to the lower chamber in the Transwell assay significantly increased (Figure 4Y,Z), confirming the chemotactic effects of THECs on BMSCs. The aforementioned results indicate that the enhanced osteogenesis‐coupling function of TS‐stimulated BMECs is attributable to the increased proportion of THECs, which aligns with the co‐existence of osteoprogenitors positive for OSX around THECs within the DO model. Together, these findings suggest a regulatory role of THECs in the DO process, potentially associated with YAP/TAZ‐mediated mechano‐biological transduction.

**Figure 5:**
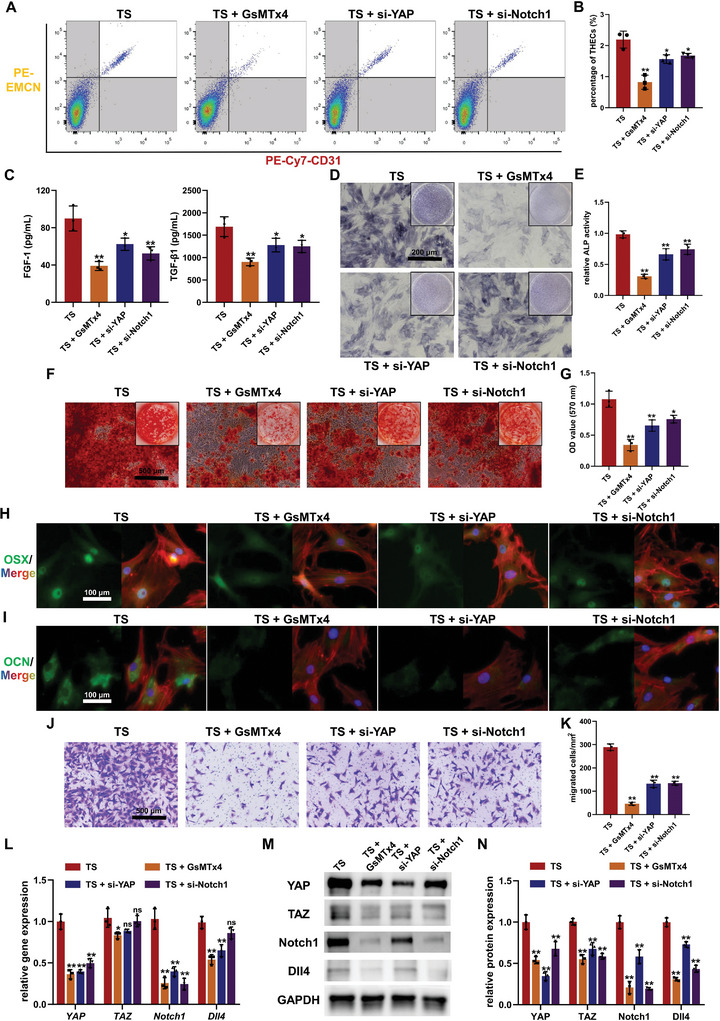
Type H endothelial cells (THECs) phenotype and expression of Notch/Dll4 and YAP/TAZ pathways after mechano‐biological transduction disruption. A,B) Flow cytometry A) and quantitative analysis B) of THECs phenotype after GsMTx4 treatment or knockdown of *YAP* or *Notch1* by siRNAs on tensile stress (TS)‐stimulated bone marrow endothelial cells (BMECs). C) Concentrations of FGF‐1 and TGF‐β1 in conditioned medium (CM) from TS‐stimulated BMECs in different treatment groups. D,E) Alkaline phosphatase (ALP) staining D) and activity measurement E) of bone marrow mesenchymal stem cells (BMSCs) after seven days of osteogenic induction with CM treatment. F,G) Alizarin red S staining F) and quantitative analysis G) of calcium nodules after fourteen days of osteogenic induction with CM treatment. H,I) Immunofluorescence staining of OSX H) and OCN I) for the evaluation of osteogenic differentiation of BMSCs. J,K) Transwell assay images J) and quantitative analysis K) revealing the chemotaxis of BMSCs in the upper chambers towards BMECs in the lower chambers. L) The gene expression of Notch1/Dll4 and YAP/TAZ pathways in TS‐stimulated BMECs upon different treatments. M,N) Western blot images M) and quantitative analysis N) revealing the expression of Notch1/Dll4 and YAP/TAZ pathways in TS‐stimulated BMSCs upon different treatments. ^ns^ *p >* 0.05, ^*^ *p <* 0.05, ^**^ *p <* 0.01.

To further elucidate the role of YAP/TAZ‐mediated mechano‐biological transduction in the activation of THECs characterized by Notch1/Dll4 under TS stimulation, GsMTx4 or *YAP* gene knockdown using siRNA (si‐YAP) was employed in conjunction with the cell stretching culture system to disrupt the mechanical sensitivity or mechano‐biological transduction signaling of BMECs (Figure S2A, Supporting Information). Additionally, siRNA targeting *Notch1* gene (si‐Notch1) was utilized as a control (Figure S2B, Supporting Information) to directly suppress THECs phenotype. Subsequent flow cytometry analysis revealed that similar to *Notch1* knockdown, both GsMTx4 application and *YAP* knockdown significantly reduced the activation of THECs induced by TS stimulation, leading to a notable decrease in the secretion of FGF‐1 and TGF‐β1 by TS‐stimulated BMECs (**Figure** **5**A–C). Thus, interfering with mechanical sensitivity using GsMTx4, inhibiting mechano‐biological transduction through si‐YAP, or directly suppressing the Notch1 pathway all significantly inhibited the osteogenesis‐coupling effect of BMECs after TS stimulation. Specifically, compared to the TS group, BMSCs treated with CM from the TS + GsMTx4, TS + si‐YAP, or TS + si‐Notch1 group exhibited significantly weakened ALP activity, reduced calcium nodule formation, and diminished expression of osteogenic markers such as OSX and OCN after osteogenic induction (Figure 5D–I). These findings indicate a pronounced inhibition of the osteogenic differentiation‐promoting effect of TS‐stimulated BMECs. Furthermore, the chemotactic effects of BMECs in these three groups were significantly impaired, as evidenced by reduced BMSCs migration to the lower chamber compared to the TS group (Figure 5J,K). Moreover, outcomes from both gene and protein expression analyses revealed that the application of GsMTx4 or si‐YAP resulted in a variable inhibition of YAP/TAZ expression levels (Figure 5L–N). Consequently, the expression levels of Notch1/Dll4 signaling and *HES1* in BMECs stimulated by TS also displayed a noticeable decrease (Figure 5L–N; Figure S3, Supporting Information), aligning with the suppressed proportion of THECs within TS‐stimulated BMECs. This suggests that, under TS stimulation, YAP/TAZ‐mediated mechano‐biological transduction plays a pivotal role in the activation of the Notch1/Dll4 signaling. Notably, a noteworthy finding emerged, revealing that direct knockdown of *Notch1* expression using si‐Notch1 led to the inhibition of YAP and TAZ expression levels (Figure 5L–N), with the exception of unaffected mRNA expression of the *TAZ* gene (Figure 5L). In consideration of prior research on the reciprocal regulation between YAP/TAZ and Notch,^[^ ^24a^ ^]^ these findings propose that in TS‐stimulated BMECs, the Notch1/Dll4 signaling, functioning as a downstream effector, may also reciprocally regulate upstream regulator YAP/TAZ. This suggests the potential formation of a positive feedback regulatory loop in TS‐stimulated BMECs, though this hypothesis unquestionably necessitates further validation through molecular experiments.

**Figure 6:**
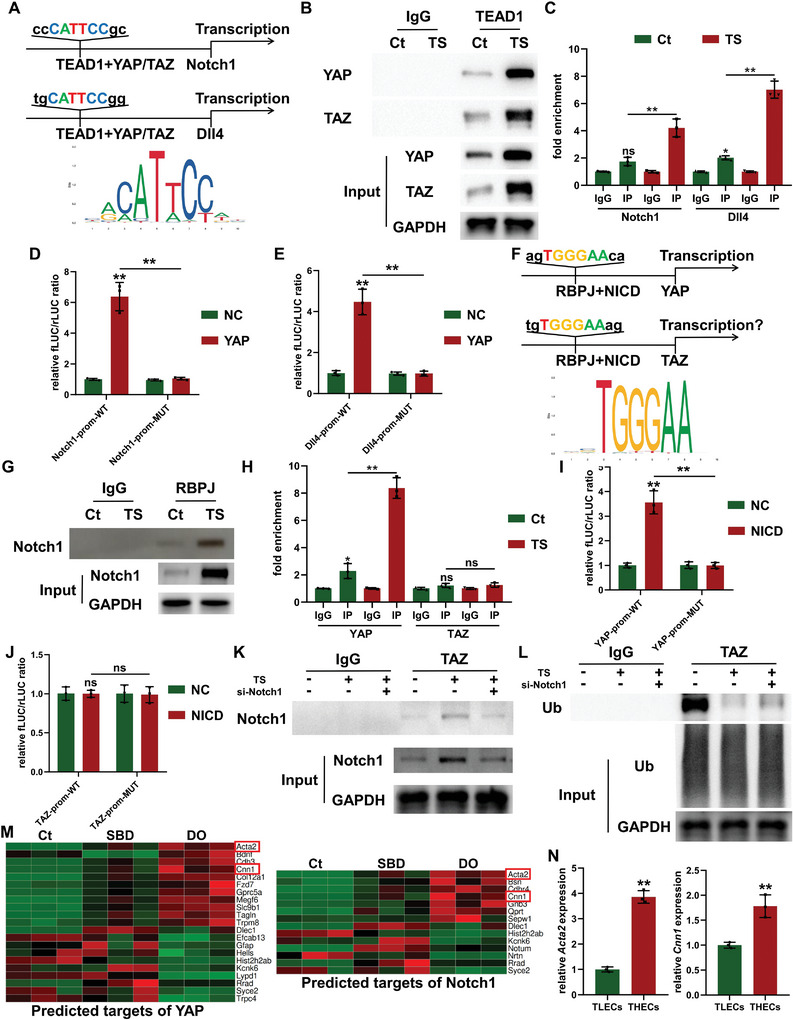
The positive feedback regulatory loop consisting YAP/TAZ and Notch1/Dll4 signaling pathways in tensile stress (TS)‐stimulated bone marrow endothelial cells (BMECs). A) Bioinformatic analyses indicating the potential binding sites of YAP/TAZ/TEAD1 in the promoters of *Notch1* and *Dll4*. B) Co‐IP assay revealing the binding of YAP/TAZ with TEAD1 as a transcriptional complex in TS‐stimulated BMECs. C) ChIP assay confirming the enhanced interaction of YAP/TAZ/TEAD1 with the predicted binding sites on promoters of *Notch1* and *Dll4* under TS stimulation. D,E) Dual‐luciferase reporter gene assays evaluate the transcriptional activity of wildtype or mutant promoters of *Notch1* D) and *Dll4* E) under the overexpression of YAP) F) Bioinformatic analyses indicating the potential binding sites of NICD/RBPJ in the promoters of *YAP* and *TAZ*. G) Co‐IP assay revealing the binding of NICD with RBPJ as a transcriptional complex in TS‐stimulated BMECs. H) ChIP assay confirming the enhanced interaction of NICD/RBPJ with the predicted binding sites on the promoter of *YAP* under TS stimulation. I,J) Dual‐luciferase reporter gene assays evaluate the transcriptional activity of wild‐type or mutant promoters of *YAP* I) and *TAZ* J) under the overexpression of NICD) K) Co‐IP assay revealing the enhanced binding of NICD to TAZ protein in BMECs under TS stimulation. L) Co‐IP assay evaluating the expression of ubiquitin (Ub) and its interaction with TAZ protein in BMECs from different groups. M) Bioinformatic analyses comparing the differentially expressed genes in different models with potential target genes of YAP and Notch1. N) The gene expression of *Acta2* and *Cnn1* in sorted type L endothelial cells (TLECs) and type H endothelial cells (THECs). ^ns^ *p >* 0.05, ^*^ *p <* 0.05, ^**^ *p <* 0.01.

YAP/TAZ, acting as transcriptional coactivators, primarily interacts with the TEAD family of transcription factors (TEAD1‐4).^[^ ^30^ ^]^ Analysis of the JASPAR database revealed a single potential binding site with a relative score greater than 0.9 between TEAD1‐4 and the *Notch1* gene promoter sequence, specifically corresponding to TEAD1 (**Figure** **6**A). Similarly, TEAD1 was found to have a potential binding site on the *Dll4* gene promoter sequence with a relative score exceeding 0.9 (Figure 6A). To authenticate the association between the envisaged transcriptional complex and the predicted binding sites, Co‐IP assays were initially conducted. The results substantiated the reciprocal interaction between YAP/TAZ and TEAD1, particularly highlighting a significant enhancement in the binding between YAP/TAZ and TEAD1 under TS stimulation (Figure 6B). Moreover, ChIP assays were executed using an anti‐YAP antibody and primers designed for the anticipated binding sites. The findings revealed a pronounced enrichment of YAP molecules at the predicted TEAD1 binding sites within the promoters of Notch1 and Dll4, which could be significantly augmented under TS stimulation (Figure 6C). Concurrently, dual‐luciferase reporter gene assays demonstrated that the overexpression of YAP (Figure S4A,B, Supporting Information) substantially elevated the expression level of fLUC in relation to rLUC under the control of the wildtype promoters of *Notch1* and *Dll4* genes (Figure 6D,E), indicating a heightened transcriptional activity of both promoters. Conversely, the overexpression of YAP had no discernible impact on the fLUC/rLUC ratio when the predicted binding sites were mutated (Figure 6D,E), providing additional confirmation of the anticipated binding sites of YAP on the promoters of *Notch1* and *Dll4* genes. These findings suggest that in TS‐stimulated BMECs, *Notch1* and *Dll4* serve as target genes of YAP/TAZ/TEAD1. This observation aligns with the increased proportion of THECs and elevated expression of Notch1/Dll4 observed in BMECs under TS stimulation, thereby furnishing supplementary experimental evidence for the activating role of YAP/TAZ‐mediated mechano‐biological transduction on Notch1/Dll4. Similarly, it has been reported that NICD primarily interacts with RBPJ to reverse its transcriptional inhibitory effect.^[^ ^31^ ^]^ According to predictions from the JASPAR database, RBPJ possesses binding sites on the promoter sequences of both *YAP* and *TAZ* genes, with relative scores greater than 0.9 (0.960 and 0.987, respectively) (Figure 6F). Furthermore, Co‐IP results confirmed the binding of NICD to RBPJ, which intensified as the expression level of Notch1 increased under mechanical TS stimulation (Figure 6G). Subsequent ChIP analysis revealed the accumulation of NICD at the predicted RBPJ binding site on the *YAP* gene promoter, rather than the promoter of *TAZ*, with even greater enrichment observed under TS stimulation (Figure 6H). Moreover, results from the dual‐luciferase reporter gene assay revealed a substantial increase in the transcriptional activity of the wildtype *YAP* gene promoter upon NICD overexpression (Figure S4C,D, Supporting Information). This was manifested by a pronounced elevation in the levels of fLUC expressed by the wildtype *YAP* promoter compared to rLUC (Figure 6I). In contrast, NICD overexpression did not significantly impact the fLUC/rLUC ratio when a binding site mutation was introduced to the *YAP* gene promoter (Figure 6I), providing additional confirmation of the anticipated binding site of NICD on the *YAP* gene promoter.

Nevertheless, enrichment of NICD on the *TAZ* gene promoter was not observed under either normal or TS stimulation conditions (Figure 6H). Additionally, overexpression of NICD did not impact the transcriptional activity of the wildtype *TAZ* gene promoter (Figure 6J). This result is consistent with previous findings that showed a negligible decrease in *TAZ* gene expression in TS‐stimulated BMECs upon si‐Notch1 application, suggesting that the reduction of TAZ protein expression is not attributed to the transcriptional regulation of NICD. Previous studies have suggested that NICD expression could influence TAZ protein degradation.^[^ ^24a^ ^]^ To further investigate this mechanism, we examined the ubiquitination levels of the TAZ protein, which is crucial for intracellular protein degradation.^[^ ^32^ ^]^ The results revealed a significant increase in the binding of NICD to TAZ following TS stimulation in BMECs, accompanied by the upregulation of Notch1 expression (Figure 6K). Conversely, there was a notable decrease in the amount of ubiquitin bound to the TAZ protein, despite no significant change in the overall level of ubiquitination (Figure 6L). Knockdown of Notch1 led to a reduction in the binding of NICD to TAZ, while simultaneously causing a significant increase in the binding of ubiquitin to the TAZ protein (Figure 6K,L). These findings propose that NICD‐dependent deubiquitination regulation in BMECs is pivotal in stabilizing the TAZ protein. This explains the observed decline in TAZ protein levels, independent of transcription, subsequent to Notch1 knockdown in TS‐stimulated BMECs. The above results suggest that in BMECs exposed to TS stimulation, the Notch1/Dll4 signaling acts not only as a functional effector regulated by YAP/TAZ to activate the THECs phenotype but also reciprocally aids in activating YAP/TAZ through transcriptional regulation and post‐translational modifications. This lends support to a positive feedback loop established between the YAP/TAZ and Notch1/Dll4 signaling pathways during the phenotypic transformation of BMECs under TS stimulation. Building upon this, a comparison between differentially expressed genes in the DO model and potential target genes of YAP and Notch1 in the hTFtarget database was conducted to predict downstream genes of this YAP/TAZ‐Notch circuit during the DO process. Among all identified genes, *Acta2* and *Cnn1*, recognized as common potential targets of YAP and Notch1 (Figure 6M), were selected as representatives for validation of expression levels in FACS‐sorted TLECs and THECs. The results exhibited a significantly elevated expression of *Acta2* and *Cnn1* in THECs compared to TLECs (Figure 6N), suggesting their potential association with the distinctive biological functions of THECs. However, further research is imperative to elucidate the specific functions of key effector genes in THECs‐mediated coupling of angiogenesis and osteogenesis, contributing to a deeper understanding of the physiological role of THECs in bone development and regeneration.

### 2.3 Exosomes Derived from TS‐Stimulated BMECs Improve the Coupling of Angiogenesis and Osteogenesis

**Figure 7:**
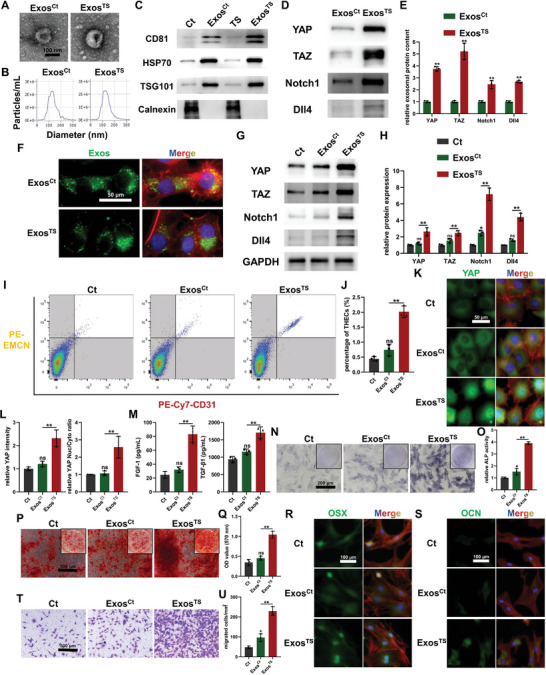
Tensile stress‐stimulated bone marrow endothelial cells (BMECs)‐derived exosomes (Exo^TS^) for transmitting the YAP/TAZ‐Notch1/Dll4 circuit to induce type H endothelial cells (THECs) phenotype. A) Transmission electron microscopy images showing the characteristic morphology of exosomes. B) Nanoparticle tracking analysis showing the distribution of particle size of exosomes. C) Western blot analysis showing the exosomal marker expression of BMECs and their exosomes in different groups. D,E) Western blot images D) and quantitative analysis E) revealing the content of exosomal proteins involved in YAP/TAZ‐Notch1/Dll4 circuit. F) Fluorescence images of BMECs after uptake of PKH67‐labeled exosomes. G,H) Western blot images G) and quantitative analysis H) revealing the expression of YAP/TAZ‐Notch1/Dll4 circuit in exosomes‐treated BMECs. I,J) Flow cytometry I) and quantitative analysis J) of THECs phenotype on exosomes‐treated BMECs. K,L) Immunofluorescence staining K) and quantitative analysis L) for evaluating the expression and nuclear translocation of YAP in exosomes‐treated BMECs. M) Concentrations of FGF‐1 and TGF‐β1 in conditioned medium (CM) from exosomes‐treated BMECs. N,O) Alkaline phosphatase (ALP) staining N) and activity measurement O) of bone marrow mesenchymal stem cells (BMSCs) after seven days of osteogenic induction with CM treatment. P,Q) Alizarin red S staining P) and quantitative analysis Q) of calcium nodules after fourteen days of osteogenic induction with CM treatment. R,S) Immunofluorescence staining of OSX R) and OCN S) for the evaluation of osteogenic differentiation of BMSCs. T,U) Transwell assay images T) and quantitative analysis U) revealing the chemotaxis of BMSCs in the upper chambers towards BMECs in the lower chambers. ^ns^ *p >* 0.05, ^*^ *p <* 0.05, ^**^ *p <* 0.01.

In the course of DO, mechanical TS predominantly occurs during the distraction phase, while effective bone regeneration, involving the participation of THECs, persists throughout the subsequent consolidation phase. This observation implies continuous maintenance and transmission of regenerative signals within the DO microenvironment, potentially encompassing the YAP/TAZ‐Notch pathway. Exosomes, serving as crucial mediators of intercellular communication by transporting various intracellular and membrane molecules, including YAP and TAZ,^[^ ^33^ ^]^ may play a pivotal role in this process. To investigate the potential of exosomes in conveying mechano‐biological transduction signaling within the DO microenvironment, exosomes were isolated from the cultured BMECs before and after TS stimulation. TEM, NTA, and western blot analyses were employed to validate their characteristic morphology, particle size, and specific marker expression (**Figure** **7**A–C). Subsequently, the molecular expression levels of the YAP/TAZ‐Notch circuit within exosomes were assessed. The results indicated that exosomes derived from BMECs after TS stimulation (Exos^TS^) exhibited elevated levels of molecular content, including YAP, TAZ, Notch1, and Dll4 (Figure 7D,E), in comparison to the control group (Exos^Ct^), aligning with the increased expression of these molecules observed in TS‐stimulated BMECs. Furthermore, co‐culture experiments employing fluorescently labeled exosomes and BMECs demonstrated the successful uptake of exosomes by BMECs, visualized through PKH67 fluorescence within the cell contours, marked with rhodamine‐labeled phalloidin, revealing a punctate distribution indicative of internalized exosomes (Figure 7F), consistent with previous studies.^[^ ^34^ ^]^ After administering exosome treatment, an assessment of molecular expression was conducted in BMECs. The outcomes revealed that Exos^Ct^ had no significant impact on the expression levels of YAP/TAZ‐Notch circuit‐associated proteins in BMECs, except for a mild upregulation of Notch1, accompanied by a slightly upregulated *HES1* expression (Figure 7G,H; Figure S5, Supporting Information). Conversely, Exos^TS^ facilitated the effective transfer of YAP/TAZ‐Notch signaling molecules to target cells, resulting in a substantial increase in the expression levels of YAP, TAZ, Notch1, Dll4, and *HES1* in BMECs within the Exos^TS^ group as compared to the Exos^Ct^ group (Figure 7G,H; Figure S5, Supporting Information). Consequently, flow cytometry analysis indicated a slight rise in the proportion of THECs in BMECs following Exos^Ct^ treatment, although the difference did not achieve statistical significance (Figure 7I,J). As anticipated, immunofluorescence staining results corroborated the molecular expression findings, exhibiting no noticeable upregulation or nuclear translocation of YAP in BMECs from the Exos^Ct^ group (Figure 7K,L). ELISA results further confirmed that the secretion levels of FGF‐1 and TGF‐β1 in BMECs treated with Exos^Ct^ remained unchanged (Figure 7M). In contrast, Exos^TS^ treatment induced a phenotypic response akin to that induced by TS stimulation, leading to a significant increase in THECs proportion, upregulation and nuclear translocation of YAP molecules, and a marked elevation in the secretion of cytokines, including FGF‐1 and TGF‐β1 (Figure 7I,M). Subsequently, the pro‐osteogenic potential of CM derived from exosome‐treated BMECs was evaluated. The results indicated that CM from the Exos^Ct^ group modestly enhanced ALP activity in BMSCs during osteogenic induction (Figure 7N,O). However, it did not exert a significant impact on calcium nodule formation and the expression of osteogenic markers such as OSX and OCN in BMSCs post‐osteogenic induction (Figure 7P–S). Additionally, the migration ability of BMSCs in the Exos^Ct^ group exhibited a mild improvement (Figure 7T,U). These outcomes suggest that the osteogenesis‐coupling function of BMECs was only slightly elevated by Exos^Ct^ treatment, likely due to the absence of a significant increase in the proportion of THECs. In contrast, CM derived from BMECs treated with Exos^TS^ significantly enhanced ALP activity, calcium nodule formation, and the expression of OSX and OCN in BMSCs during osteogenic induction (Figure 7N–S). Moreover, a greater number of BMSCs were attracted to the lower chamber of the Transwell by BMECs treated with Exos^TS^ (Figure 7T,U), collectively indicating an enhanced osteogenesis‐coupling function compared to the Exos^Ct^ group. These findings suggest that, following activation of the YAP/TAZ‐Notch positive feedback loop by TS stimulation, BMECs not only undergo a phenotypic transformation into THECs but also possess the potential to transmit regenerative signals through exosomes, contributing to efficient bone regeneration during the consolidation phase. However, it is noteworthy that the physiological regulatory role of BMECs in this hypothesis still lacks more in‐depth in situ studies for validation.

**Figure 8:**
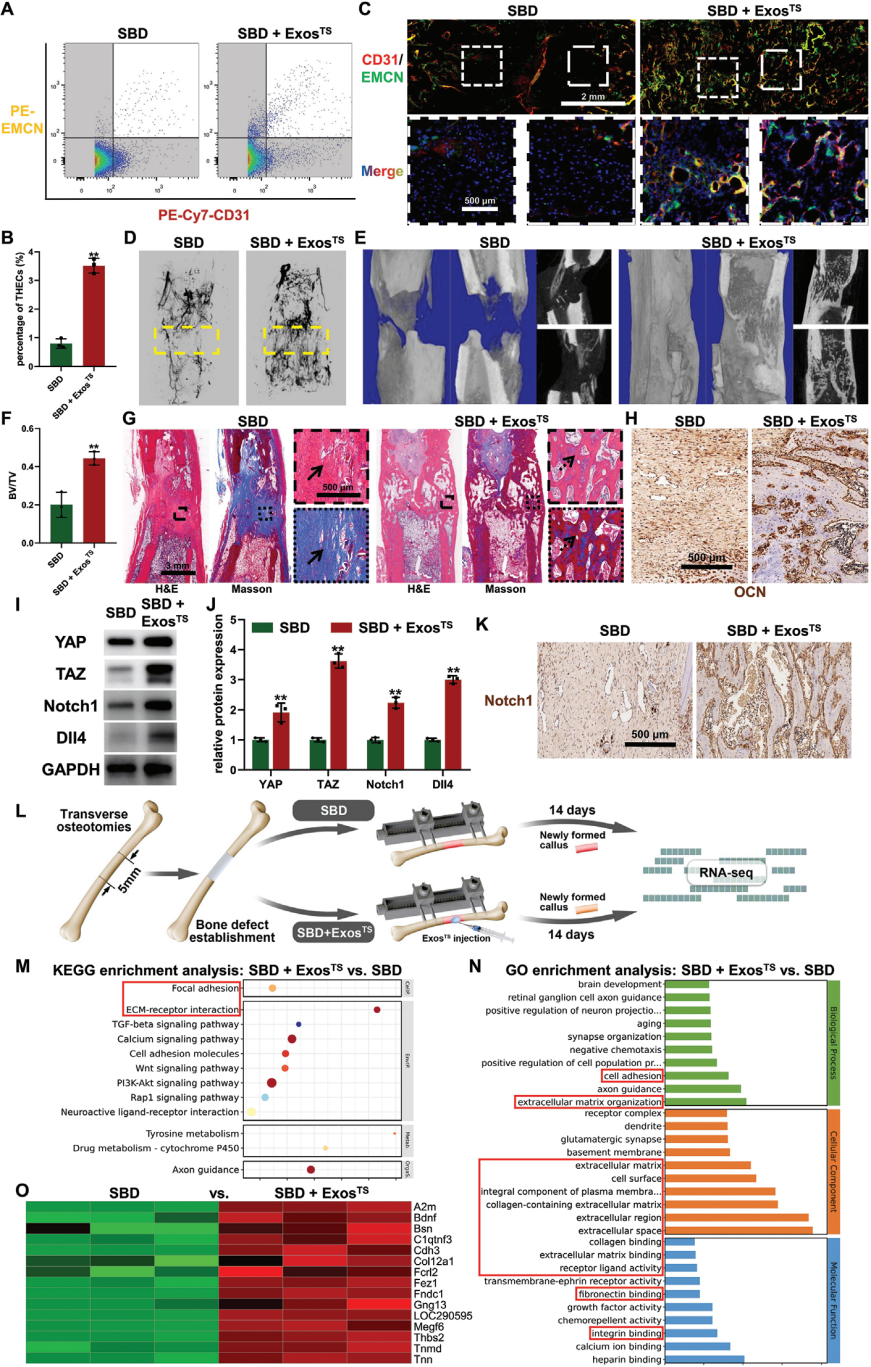
Tensile stress‐stimulated bone marrow endothelial cells‐derived exosomes (Exo^TS^) for the regeneration of segmental bone defect (SBD). A,B) Flow cytometry A) and quantitative analysis B) of THECs abundance within SBD healing area at three weeks post‐surgery. C) Immunofluorescence staining of CD31 and EMCN for the detection of THECs distribution within SBD healing area at three weeks post‐surgery. D) Images of three‐dimensional reconstruction based on the micro‐CT data collected after the vascular perfusion at three weeks post‐surgery. E,F) Images of three‐dimensional reconstruction (left) and coronal and sagittal sections (right) E) and bone volume/tissue volume (BV/TV) quantitative analysis F) based on the micro‐CT data collected at five weeks post‐surgery. G) H&E (left, top) and Masson (right, down) staining of the regenerated callus at five weeks post‐surgery. H) Immunohistochemical staining of OCN in the healing area of SBD at five weeks post‐surgery. I,J) Western blot images I) and quantitative analysis J) revealing the expression YAP/TAZ‐Notch1/Dll4 circuit in the regenerated callus of SBD at three weeks post‐surgery. K) Immunohistochemical staining of Notch1 in the healing area of SBD at three weeks post‐surgery. L) Schematic representation of transcriptomic sequencing comparing the SBD and the SBD + Exo^TS^ groups. M,N) GO enrichment analysis M) and KEGG enrichment analysis N) of upregulated genes in the regenerated tissue during SBD healing after Exo^TS^ therapy indicating activated cell‐matrix signals (red rectangle). O) Heat map of identical differentially expressed genes involved in cell‐matrix signals in healing callus of Exo^TS^‐treated SBD and distraction osteogenesis, compared to SBD group. ^**^ *p <* 0.01.

Given that Exos^TS^ partially reproduces the effects of TS stimulation on BMECs in vitro, it is speculated that pre‐cultivation under TS can be considered a promising modification strategy to enhance the therapeutic efficacy of exosomes derived from BMECs for SBD biological treatment. To validate the hypothesized promoting effect of Exos^TS^ on the activation of THECs for segmental bone regeneration, fluorescently labeled exosomes were initially administered into the bone defect area, and their stable distribution in the target region was confirmed a week post‐injection through IVIS detection (Figure S6, Supporting Information). Three weeks post‐surgery, flow cytometric analysis of the regenerated tissue in the bone defect area showed a significant increase in the local abundance of THECs with the application of Exos^TS^ (**Figure** **8**A,B). Immunofluorescence double staining of CD31/EMCN further supported these findings by demonstrating a significant increase in THECs abundance within the bone defect area following stereotactic injection of Exos^TS^, although they did not form the metaphysis‐like architecture (Figure 8C). Additionally, Exos^TS^ treatment significantly improved overall neovascularization within the regeneration area (Figure 8D). Similarly, micro‐CT examination revealed that Exos^TS^ treatment greatly enhanced local bone tissue regeneration and mineralization, resulting in continuous cortical bone and a substantial increase in BV/TV at five weeks post‐surgery, compared to the SBD group (Figure 8E,F). The histological analysis provided further support for these results, as evidenced by the presence of abundant newly formed bone trabeculae in the regeneration area of the SBD + Exos^TS^ group, as observed in H&E and Masson staining (Figure 8G). In contrast, the SBD group exhibited a large amount of remaining unmineralized fibrous tissue (Figure 8G). Furthermore, the regeneration area in the SBD + Exos^TS^ group showed a more pronounced expression of OCN, suggesting enhanced osteogenic activity due to Exos^TS^ treatment (Figure 8H). These findings unequivocally demonstrate the remarkable therapeutic effect of Exos^TS^ on SBD, even without the application of the Ilizarov technique. Additionally, the regenerated callus of the SBD + Exos^TS^ group, as expected, exhibited higher expression levels of YAP, TAZ, Notch1, and Dll4 proteins compared to the SBD group, and immunohistochemical analysis also indicated that Exos^TS^ significantly upregulated the expression of Notch1 within the local defect area (Figure 8I–K), indicating that the promoting effect of Exos^TS^ on segmental bone regeneration is associated with the transmission of activated YAP/TAZ‐Notch circuit‐related molecules. To comprehensively understand the biological therapeutic effects of Exos^TS^ on SBD, transcriptome sequencing was applied to the regenerative tissue of the SBD and SBD + Exos^TS^ groups, revealing significant enrichment of upregulated differentially expressed genes enriched in cell‐matrix signals in the SBD + Exos^TS^ group (Figure 8L–N). This observation was substantiated by both GO enrichment analysis and KEGG enrichment analysis, intriguingly, with 15 specific genes precisely matching the upregulated genes identified in cell‐matrix signals activated during the DO process (Figure 8M–O). This suggests that Exos^TS^ therapy, as a biological therapeutic approach inspired by the Ilizarov technique, mimics, to some extent, the regenerative effects of TS stimulation on SBD.

## 3 Discussion

**Figure 9:**
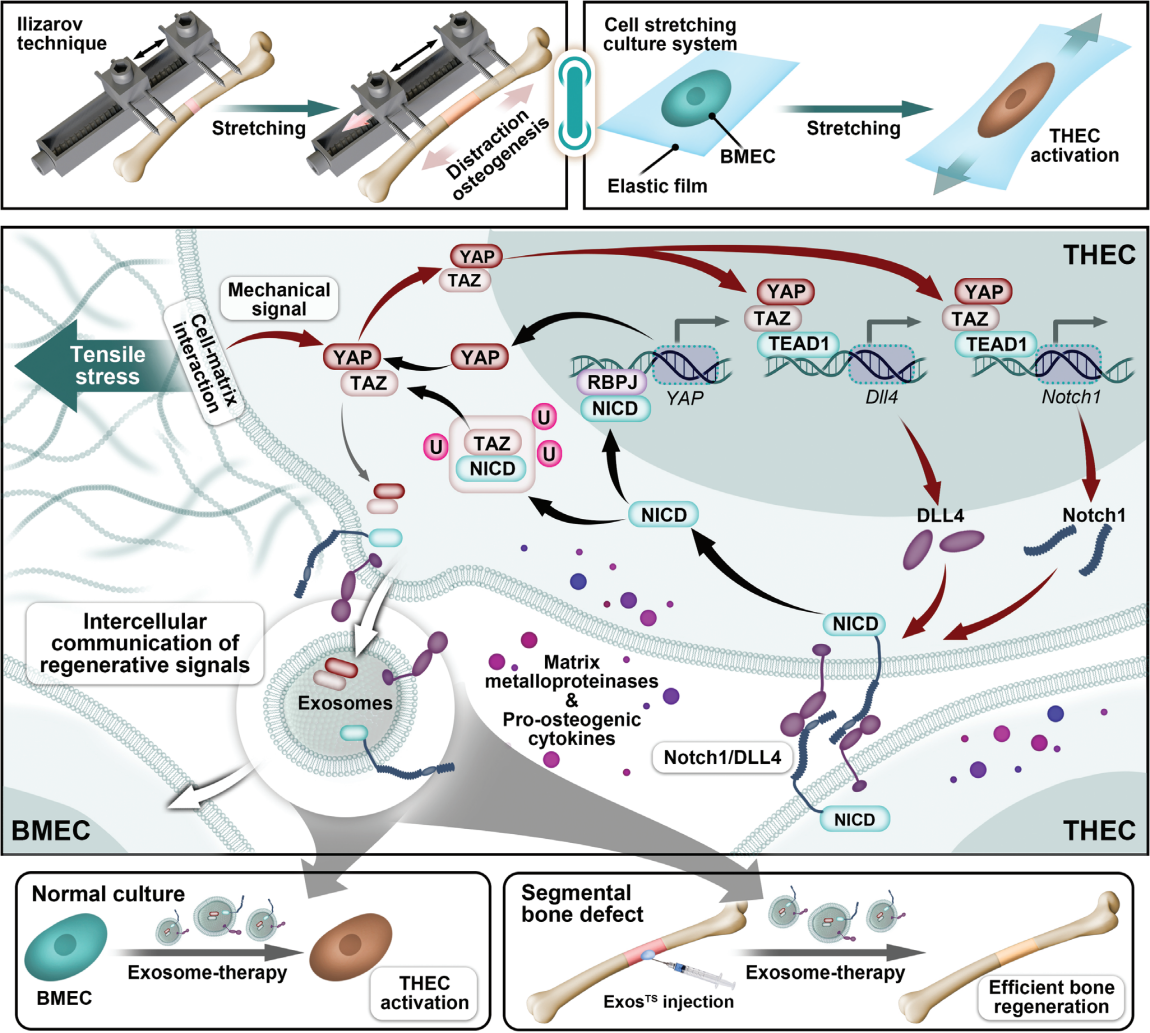
Schematic representation of tensile stress‐induced activation of type H endothelial cell (THEC) during distraction osteogenesis (DO). During the Ilizarov technique‐induced DO process, mechanical tensile stimulation initiates the activation of YAP/TAZ, an essential transcriptional coactivator involved in mechano‐biological transduction signaling, through cell‐matrix interactions in bone marrow endothelial cell (BMEC). Subsequently, YAP/TAZ collaborates with TEAD1 to bind to the promoters of *Notch1* and *Dll4* genes, promoting their transcription and activating the crucial Notch signaling for THEC activation. Concurrently, the NICD collaborates with RBPJ to enhance *YAP* gene transcription by binding to its promoter, and inhibits ubiquitin‐mediated degradation of TAZ protein, reciprocally assisting in activating YAP/TAZ signaling. Furthermore, the key molecules involved in this positive feedback loop can be effectively transported by exosomes, potentially contributing to the intercellular communication of regenerative signals, which holds a promising perspective as a biotherapeutic strategy inspired by Ilizarov technique for activating THEC and treating segmental bone defect.

While the Ilizarov technique has been widely employed in orthopedics and traumatology for over three decades to promote segmental neovascularization and bone regeneration,^[^ ^35^ ^]^ its underlying mechanism and contribution to the coupling of angiogenesis and osteogenesis remain unclear. Additionally, the potential involvement of THECs in bone regeneration has only recently gained recognition, despite extensive investigation of their role as key regulators in developmental biology.^[^ ^36^ ^]^ This study aims to bridge the gap between clinical application and theoretical understanding by conducting a comprehensive analysis of gene expression patterns and endothelial properties using a rat DO model and a cell tension system. Our findings provide evidence that TS triggers YAP/TAZ‐mediated mechano‐biological transduction signaling, resulting in the activation of Notch1/Dll4 for THECs‐mediated coupling of angiogenesis and osteogenesis both in vivo and in vitro. Moreover, through the targeted disruption of mechano‐biological transduction signaling in BMECs and a comprehensive series of biomolecular interaction analyses, we elucidate a specific transcriptional activation effect of YAP/TAZ/TEAD1 on Notch1 and Dll4. This activation instigates the phenotypic transition towards THECs in response to TS, concomitantly accompanied by an upregulation of NICD that positively reinforces YAP transcription through RBPJ and inhibits TAZ ubiquitination. Importantly, this positive feedback signaling cascade can be propagated via exosomes, thereby imparting additional therapeutic benefits (**Figure** **9**).

Prior investigations have demonstrated an augmented expression of genes associated with the activator protein 1, Wnt, and focal adhesion kinase pathways, which play pivotal roles in embryonic bone development, throughout the DO process.^[^ ^37^ ^]^ This observation implies a potential parallelism between the DO process and bone development; however, the absence of a definitive theoretical foundation and experimental substantiation warrants further exploration. It has been documented that cell‐matrix signals, exemplified by *Itgb1* in endothelial cells, significantly contribute to maintaining the normal structure and function of THECs located in the metaphysis, which orchestrate bone development through proteolytic and pro‐osteogenic functions.^[^ ^14^, ^15^, ^18^ ^]^ Our findings underscore a comparable enrichment of cell‐matrix adhesion molecules in the regenerated tissue of the DO process in comparison to the SBD model. Furthermore, considering the essential role of cell‐matrix interactions in activating mechano‐biological transduction signaling, as reported to contribute to TS‐induced osteogenic differentiation of BMSCs,^[^ ^12^ ^]^ it is reasonable to hypothesize that THECs may exert regulatory influences on bone generation, akin to their role in bone development, during the DO process. In our present investigation, we observed the formation of a metaphysis‐like structure comprised of columnar tubes and arches by THECs on both sides of the distraction area. This coincided with a heightened expression of MMP9 and the presence of surrounding osteoprogenitors positive for OSX. The conspicuous anatomical and functional parallels between THECs in the DO process and bone development establish a novel biological foundation supporting the proposition that the Ilizarov technique elicits a regenerative potential for bone akin to skeletal development. Moreover, the application of the mechano‐sensitive ion channel inhibitor GsMTx4 or the endothelial cell‐specific YAP knockdown, aimed at disrupting BMECs’ mechanical sensitivity or mechano‐biological transduction signaling, led to a decrease in THECs abundance and compromised bone regeneration during the DO process. Therefore, we deduce that mechanical tension conveyed through cell‐matrix interactions, specifies the THECs subpopulation potentially via downstream YAP/TAZ‐mediated mechano‐biological transduction signaling. Certainly, the process of cell mechanotransduction is intricate, encompassing complexities such as nuclear mechanosensing‐dependent chromatin remodeling, which may occur simultaneously with the activation of YAP/TAZ, influencing transcriptional activity.^[^ ^38^ ^]^ Consequently, the comprehensive biological mechanisms underlying the effects of TS are still in need of further refinement. Moreover, the prevailing activation profile of cell‐matrix signals in regenerated tissue of DO process demonstrates a primary upregulation of genes encoding adhesion molecules such as *Fndc1*, *Tnn*, *Cdh3*, and others—findings heretofore unreported in previous studies focusing on THECs during bone development.^[^ ^18a^ ^]^ Further experiments are imperative to elucidate the distinct contributions of specific adhesion molecules during both the DO process and skeletal development.

Notch1/Dll4 represents the critical characteristic signal in THECs to activate the coupling of angiogenesis and osteogenesis during skeletal development.^[^ ^18b^ ^]^ Additionally, inducible Notch activation has been reported to enhance angiogenesis and promote bone mineralization in the early consolidation phase of DO.^[^ ^39^ ^]^ Our research further confirms that the upregulation of THECs abundance during the DO process, compared to the SBD model, is associated with the activation of the Notch1/Dll4 pathway. However, these findings contradict the Notch‐mediated suppression of neovascularization observed in most organs and malignant tumors,^[^ ^19^ ^]^ and the mechanisms behind the distinct pro‐angiogenic role of Notch signaling in bone metabolism, diverging from its role in other microenvironments, remain poorly understood. Interestingly, the regulatory relationship between YAP/TAZ and Notch signaling also exhibits significant context‐dependence across various physiological processes. In the differentiation of epidermal progenitors, YAP overexpression leads to a reduction in Notch transcriptional responses, and conditional YAP/TAZ knockout triggers Notch signaling.^[^ ^23^ ^]^ Simultaneously, in the epithelium, the loss of Notch triggers the activation of YAP/TAZ.^[^ ^40^ ^]^ Consequently, in the skin, the inhibition of Dll4/Notch signaling has been reported to promote functional angiogenesis.^[^ ^41^ ^]^ Conversely, in liver growth and the DO process under investigation in this study, YAP/TAZ and Notch signaling form positive feedback loops, and the liver and bones happen to be the only two organs where the Notch signaling‐dependent THECs reside.^[^ ^14^, ^24^ ^]^ These preceding and current research results collectively suggest a potential connection between the regulatory role of Notch signaling in angiogenesis and its interaction with YAP/TAZ in different contexts. Notably, the transcriptional coactivator YAP/TAZ, responsible for mediating mechano‐biological transduction signaling, has highly conserved functions in promoting angiogenesis through the activation of hypoxia‐inducible factor 1α and vascular endothelial growth factor signaling, which are distinctive characteristics of THECs as well.^[^ ^14^, ^42^ ^]^ Therefore, it is likely that the distinctive pro‐angiogenic role of Notch signaling in bone can be at least partially attributed to the mutually reinforcing regulatory network it forms with YAP/TAZ in the skeletal context. Admittedly, the results of our study are far from sufficient to confirm this conclusion. However, our direction for further investigation is promising, focusing on the screening of potential target genes that show differential expression between the DO process and the SBD model concerning YAP/TAZ and Notch. Of particular interest are the common high expression levels of the target genes *Acta2* and *Cnn1* in THECs compared to TLECs, both of which have been reported to contribute to angiogenesis.^[^ ^43^ ^]^ Moreover, additional research is imperative to explore potential relationships between YAP/TAZ‐Notch regulatory patterns and the regulatory role of Notch signaling in angiogenesis across various tissue microenvironments.

The distraction phase of DO provides transient TS stimulation, while the metaphysis‐like architecture based on THECs, as discovered in this study, remains persistently active, promoting efficient bone regeneration in the subsequent prolonged consolidation phase, during which the mechanical tensile strain within the distracted tissue gradually diminishes. One possible explanation for the duration of THECs activation independent of TS stimulation is that initially activated regenerative signals could be efficiently maintained and transmitted, akin to the inflammatory cascade observed during injury recovery, sepsis, and infections.^[^ ^44^ ^]^ While the positive feedback loop between YAP/TAZ and Notch1/Dll4 signaling provides a biological basis for synergistically activating tissue regeneration signals, intercellular communication of regenerative signals within the microenvironment is also crucial. Exosomes serve as mediators of intercellular communication primarily by transporting proteins and small RNAs, and these bioactive agents are typically highly expressed in the cells they originate from.^[^ ^45^ ^]^ Our findings demonstrate that the molecules associated with the YAP/TAZ‐Notch1/Dll4 circuit are similarly enriched in exosomes derived from TS‐stimulated BMECs. Moreover, the administration of these exosomes can effectively activate THECs phenotype in both quiescent BMECs in vitro and the SBD area in vivo, at least partially simulating the physiological abundance of THECs and the activation of cell‐matrix signals in the DO process. Therefore, we speculate that exosomes derived from TS‐stimulated BMECs may play the physiological role of transmitting the positive feedback regenerative signal in response to mechanical stimulation, thereby maintaining the efficiency of bone regeneration during the consolidation phase. Moreover, to the best of our knowledge, this is the first study to potentially translate the molecular mechanism of the Ilizarov technique into a biotherapy approach for SBD treatment.

## 4 Conclusion

In summary, our data highlight the crucial role of TS‐induced mechano‐biological transduction signaling in regulating THECs‐mediated coupling of angiogenesis and osteogenesis, particularly during DO process. Activation of YAP/TAZ signaling, in response to TS stimulation, establishes a positive feedback loop with Notch1/Dll4 that governs the THECs phenotype. Numerous molecules involved in this endothelial YAP/TAZ‐Notch circuit are packaged within exosomes derived from TS‐stimulated BMECs, potentially facilitating intercellular communication within the DO microenvironment. Exploiting these exosomes holds significant promise for enhancing the healing of SBD by mimicking the therapeutic effects of Ilizarov technique on THECs‐mediated coupling of angiogenesis and osteogenesis. These findings not only advance our understanding of TS‐induced segmental bone regeneration but also provide a foundation for innovative translational therapeutic strategies inspired by the underlying biological mechanisms of the Ilizarov technique.

## 5 Experimental Section

### Animal Surgery and Treatment

All animal procedures were approved by the Animal Research Committee of Shanghai Sixth People's Hospital Affiliated with Shanghai Jiao Tong University School of Medicine (DWSY2022‐0079). Male SPF Sprague‐Dawley (SD) rats, aged 10 to 12 weeks and weighing approximately 400 g, were utilized in this study, with 15 to 21 individuals randomly assigned to each experimental group. After anesthesia, skin preparation, and exposure, two transverse osteotomies were performed at the midshaft of the right tibia, spaced 5 mm apart. To establish the SBD model, customized unilateral external fixators (Xinzhong, China) were applied to stabilize the proximal and distal bone segments on both sides of the 5‐mm defect. In order to establish the DO model, the bone segments adjacent to the defect were aligned using the external fixator. Subsequently, the incision was closed layer‐wise. The Ilizarov technique was employed to induce DO process, consisting of three postoperative stages: a four‐day latency phase, a ten‐day distraction phase with a rate of 0.25 mm every 12 h, and a three‐week consolidation phase. GsMTx4 (2 µM, 50 µL; HY‐P1410, MCE) treatment was administered during the distraction phase on days 0 and 5 through stereotactic injection into the distraction zone, while an equal volume of PBS solution served as the control. For the exosome therapy of SBD, exosomes (50 µg mL^−1^, 50 µL) derived from TS‐stimulated BMECs were stereotactically injected into the bone defect area every two weeks following the surgery, with an equal volume of PBS solution used as a control.

### RNA‐seq Analysis

Regenerated tissue from the DO model and SBD model was used for transcriptome sequencing to gain preliminary insight into the mechanistic differences between DO process and conventional bone regeneration. In the DO model, the tissue from the distraction zone was collected immediately after the distraction phase. The regenerating tissue from the SBD model was collected two weeks post‐surgery. As a control, tissue from the midshaft of the normal tibia, obtained during animal modeling, was included. To delve into the underlying mechanisms through which exosome therapy enhances bone regeneration, tissue samples were collected from both the SBD group and the SBD + Exos^TS^ group two weeks after the surgical procedure. RNA extraction, RNA sequencing, and bioinformatics analysis were performed on the newly formed callus of all groups with the assistance of Shanghai OE Biotech Co., Ltd (China).

### Adeno‐Associated Virus (AAV) Targeting Transduction

In order to achieve targeted YAP knockdown in the endothelial cells within the bone regeneration area for interference with their mechanical signaling, serotype ENT AAV was selected as the transfection vector. Simultaneously, a short hairpin RNA targeting rat *YAP* (sh‐YAP) or a negative control sequence was inserted into the TIEp‐MCS (sh‐YAP)‐WPRE‐SV40 PolyA vector, which was expected to further ensure the specificity of the obtained AAV (AAV‐sh‐YAP) for in vivo transfection of BMECs, utilizing the endothelial cell‐specific promoter. Serotype ENT AAV carrying TIEp‐EGFP‐MCS‐WPRE‐SV40 PolyA was employed for the visualization of targeted in vivo transfection of CD31‐positive BMECs. To selectively interfere with endothelial mechanical signals in the DO model, the control AAV or AAV‐sh‐YAP (50 µL, 2×10^11^ V.G mL^−1^) was stereotactically injected into the osteotomy site on the first‐day post‐surgery. The sequence of sh‐YAP targeting rat *YAP* (5′‐GCTGCCACCAAGTT‐3′) and all AAV constructs used in this study were synthesized and constructed by GeneChem (China).

### Micro‐Computed Tomography (Micro‐CT)

After the three‐week consolidation phase, tibial samples from the DO models in each group were harvested, while tibial samples from all SBD models were collected at five weeks post‐surgery. Micro‐CT scanning (Bruker, Germany) with a slice thickness of 18 µm was used to assess bone regeneration and mineralization. The CT data were reconstructed in three dimensions using CTVox software (Bruker), and coronal and sagittal images were obtained using DataViewer software (Bruker). Bone volume/tissue volume (BV/TV) in the bone defect or distraction zone was calculated using CTAn software (Bruker). Additionally, at three weeks post‐surgery, rats from the SBD group and SBD + Exos^TS^ group underwent Microfil (MV‐122, Flow‐Tech) perfusion, followed by decalcification of tibial samples. Subsequently, micro‐CT scanning (Bruker) with a slice thickness of 9 µm and three‐dimensional reconstruction was performed to evaluate vascular neogenesis in the defect area.

### Histological and Immunofluorescence Assays

Tibial samples from the DO models in each group were collected after the three‐week consolidation phase, while samples from the SBD models were obtained at five weeks post‐surgery. Subsequently, the tibial samples were fixed in 4% (w/v) paraformaldehyde (PFA) for 24 h, decalcified in 10% (w/v) EDTA for three weeks, dehydrated using a graded alcohol series, and finally embedded in paraffin for 5 µm‐thick sections. To evaluate bone regeneration in the distraction zone or defect area through histological analysis, paraffin sections were subjected to H&E staining (G1120, Solarbio) and Masson's trichrome staining (G1346, Solarbio) following the manufacturer's instructions. For immunohistochemical staining, tibial samples were collected three or five weeks after surgery for paraffin embedding as described above to detect the expression of Notch1 or osteocalcin (OCN), respectively. The paraffin sections underwent antigen retrieval and blocking, followed by overnight incubation with anti‐Notch1 antibody (1:500; #3608, CST) or anti‐OCN antibody (1:200; A6205, ABclonal) at 4 °C. After incubation with the horseradish peroxidase (HRP)‐conjugated secondary antibody (1:5000; 111‐035‐003, Jackson ImmunoResearch) for 2 h at room temperature, the sections were visualized using an HRP‐streptavidin system.

CD31/EMCN and MMP9/OSX immunofluorescence double staining were employed to evaluate the distribution and function of THECs in the distraction zone and bone defect area. Tibial samples from all DO models were collected one week after the completion of distraction, while samples from the SBD models were obtained three weeks post‐surgery. Subsequently, tibial samples were decalcified using 18% (w/v) EDTA for one week, dehydrated in 30% (w/v) sucrose solution for 24 h, embedded in OCT reagent, and finally sliced into 10 µm‐thick sections. After blocking, the sections were incubated overnight at 4 °C with primary antibodies, followed by incubation with fluorescent‐labeled secondary antibodies (1:200; ab97035, ab6840, ab150077, Abcam) at room temperature for 2 h. 4′,6‐diamidino‐2‐phenylindole (DAPI; Servicebio, G1012) was used to stain the cell nuclei, and the sections were observed under a DMi8 fluorescence microscope (Leica, Germany). The primary antibodies utilized in this study included anti‐CD31 (1:100; ab64543, abcam), anti‐EMCN (1:100; sc‐65495, Santa Cruz), anti‐MMP9 (1:500; sc‐393859, Santa Cruz), anti‐OSX (1:500; ab209484, abcam).

### Cell Culture and Treatment

Human bone marrow endothelial progenitor cells (CP‐H162, Procell) were seeded onto fibronectin‐precoated culture dishes and cultured in EGM‐2 BulletKit (CC‐3162, Lonza) with passage every three days for the maturation of BMECs. Passage 5 to 10 cells were used for subsequent treatments and analysis. To apply mechanical TS to the cells, BMECs were seeded onto protection‐coated flex cell plates (FlexCell, USA) and subjected to cyclic stretching at 10% amplitude and 0.5 Hz frequency for 24 h. To inhibit the mechanical sensitivity of BMECs, GsMTx4 (2 µM) was added to the cell culture medium 2 h prior to the application of mechanical stretching stimulus. To suppress YAP and Notch1 expression, siRNAs targeting the *YAP* or *Notch1* were transfected into the BMECs using RNAiMAX (13778100, Invitrogen) 24 h before the application of mechanical stretching stimulus, with si‐NC (A06001, GenePharma) used as a control. The gene‐specific siRNAs were synthesized by GenePharma (China), and the sequences were as follows: YAP siRNA: 5′‐CUGCCACCAAGCUAGAUAATT‐3′; Notch1 siRNA: 5′‐GCACGCGGAUUAAUUUGCATT‐3′. To perform exosome therapy, BMECs were seeded in six‐well plates and cultured until 80% confluence. Then the culture medium was replaced with EGM‐2 medium supplemented with exosomes from each group (50 µg mL^−1^), and the cells were further cultured for 48 h for subsequent analysis. Human bone marrow mesenchymal stem cells (BMSCs; CP‐H166, Procell) were cultured in Minimal Essential Medium Alpha (SH30265.01, HyClone) supplemented with 10% fetal bovine serum (10099‐141, Gibco) and 1% penicillin‐streptomycin (15140122, Gibco). The passage was performed every three days, and BMSCs from the second to fifth passages were used for subsequent osteogenic differentiation and migration experiments.

### Flow Cytometry

To assess the presence of tibial THECs within the bone regeneration area, tibial samples were collected from the DO models one week after the completion of distraction, as well as from the SBD models three weeks post‐surgery. Fresh callus was crushed in sterile PBS devoid of calcium and magnesium ions and maintained at a cold temperature. Subsequently, the callus was digested in collagenase A (10103578001, MCE) for 30 min at 37 °C and filtered through a 40‐µm mesh to obtain a single‐cell suspension. After blocking with 5% (w/v) BSA (SW3015, Solarbio), the resulting cell suspensions were incubated at room temperature with APC‐CD45 (1:100; 17‐0461‐82, eBioscience), FITC‐Ter119 (1:100; MA5‐17580, eBioscience), PE‐Cy7‐CD31 (1:100; 25‐0310‐82, eBioscience), and PE‐EMCN (1:100; sc‐65495 PE, Santa Cruz) antibodies for 45 min. Flow cytometry analysis was then performed to evaluate the cell populations. Within the CD45‐Ter119‐CD31+ population of total BMECs, cells expressing high levels of CD31 and EMCN were identified as THECs. To determine the proportion of THECs within BMECs following mechanical TS or exosome therapy, treated BMECs were incubated with PE‐Cy7‐CD31 (1:100; 25‐0319‐42, eBioscience) and PE‐EMCN (1:100; 12‐5851‐82, eBioscience) antibodies at room temperature for 45 min after blocking with 5% (w/v) BSA. Cells expressing high levels of CD31 and EMCN were recognized as THECs through flow cytometry analysis (BD Biosciences, USA). To isolate TLECs and THECs from TS‐stimulated BMECs, the treated cells were similarly co‐incubated with the aforementioned fluorescently labeled anti‐CD31 and anti‐EMCN antibodies. Subsequently, the labeled BMECs were subjected to fluorescence‐activated cell sorting (FACS; BD Biosciences). THECs, characterized by high expression of CD31 and EMCN in the upper right quadrant, and TLECs, characterized by low expression of CD31 and EMCN in the lower left quadrant, were sorted and individually seeded onto fibronectin‐precoated culture dishes for subsequent cultivation and analysis.

### Preparation of Conditioned Medium (CM) and Cytokine Detection

Following mechanical TS or exosome therapy, the culture medium of BMECs was replenished and continued to be cultured for an additional 48 h. Likewise, the sorted TLECs and THECs were cultured until reaching 80% confluence. Subsequently, the culture medium was replaced, and the cells were further incubated for 48 h to collect CM. Subsequently, the CM from each group was centrifuged at 2000 g for 10 min to obtain the supernatant. To measure the concentrations of FGF‐1 and TGF‐β1 in the CM, an enzyme‐linked immunosorbent assay (ELISA) was performed using commercial kits (CSB‐E04546h, CSB‐E04725h, CUSABIO) according to the manufacturer's instructions. In brief, 100 µL of the sample, 100 µL of biotinylated antibody, and 100 µL of HRP‐avidin were sequentially added and incubated in the assay plate. Then, 90 µL of TMB substrate was added to each well and incubated at 37 °C for 20 min. The reaction was stopped by adding a stop solution, and the OD value was measured at 450 nm. The cytokine concentrations were calculated based on the standard curve.

### Osteogenic Differentiation

To investigate the impact of CM derived from different groups of BMECs on the osteogenic differentiation of BMSCs, alkaline phosphatase (ALP) activity and calcium nodule formation were assessed. Briefly, BMSCs were seeded in 24‐well plates at a density of 50 000 cells per well. Once the cells adhered and reached 60–80% confluence, the culture medium was replaced with osteogenic induction medium (HUXMX‐90021, OriCell) supplemented with 20% CM from BMECs. The medium was changed every two days. For ALP staining, BMSCs after 7 days of osteogenic induction were fixed, washed, and incubated with BCIP/NBT staining solution (C3206, Beyotime) for 30 min. To measure ALP activity, BMSCs after 7 days of osteogenic induction were lysed, and the samples were incubated with para‐nitrophenyl phosphate (P0321S, Beyotime) for 10 min, followed by measuring the absorbance at 405 nm. On the 14th day of osteogenic differentiation, calcium nodules were stained with alizarin red S solution (ALIR‐10001, OriCell), and mineralization was quantitatively analyzed by dissolving the calcium nodules with 10% (w/v) cetylpyridinium chloride and measuring the absorbance at 570 nm.

### Immunocytochemistry Assay

To assess the osteogenic differentiation of BMSCs in each group, after seven days of osteogenic induction, cells were fixed with 4% (w/v) PFA and permeabilized using 0.1% (v/v) Triton X‐100 in PBS. Following a 1‐hour blocking step with 5% (w/v) BSA, the cells were incubated overnight at 4 °C with anti‐OSX antibody (1:500; ab209484, abcam) or anti‐OCN antibody (1:200; A6205, ABclonal). Similarly, to evaluate the YAP expression levels and intracellular localization of TLECs and THECs sorted from the groups and the BMECs treated with exosomes, cells were fixed, permeabilized, and blocked. Subsequently, they were incubated with an anti‐YAP antibody (1:100; A19134, ABclonal) overnight at 4 °C. The cells were then washed three times with PBS and incubated with an Alexa Fluor 488‐conjugated secondary antibody (1:500; ab150077, Abcam) for 1 hour. Afterward, the cells were stained with TRITC‐conjugated phalloidin (100 nM; Yeasen, 40734ES75) and DAPI (2 µg mL^−1^; Servicebio, G1012) for 15 min to respectively visualize F‐actin and cell nuclei. The fluorescence signal was captured using a DMi8 fluorescence microscope (Leica).

### Transwell Assay

BMSCs were suspended in blank culture medium and seeded (20 000 cells per well) in the upper chambers of Transwell plates with 8‐µm pore size (Corning, USA). Sorted TLECs, THECs, or treated BMECs in each group were re‐seeded to the lower chambers (50 000 cells per well), and the cells were co‐incubated for 12 h. Subsequently, non‐migratory cells in the upper chambers were removed by gently wiping with a cotton swab, and the migrated BMSCs in the lower chambers were fixed and stained with crystal violet solution (C0121, Beyotime).

### Quantitative Real‐Time Polymerase Chain Reaction (qPCR) Analysis

After the completion of mechanical TS stimulation, total RNA from the different groups of BMECs was extracted using an EZ‐press RNA Purification Kit (B0004D, EZBioscience). Subsequently, 500 ng of total RNA was utilized to synthesize cDNA using a Reverse Transcription Kit (A0010, EZBioscience), followed by qRT‐PCR analysis using SYBR Green qPCR Master Mix (A0012, EZBioscience). The gene relative expression levels were calculated using the 2^−ΔΔCT^ method based on the CT values obtained from each group, with GAPDH serving as the reference gene. The primers were synthesized by GenePharma (China), and the sequences are listed in Table S1 (Supporting Information).

### Western Blot

Tibial samples from the DO models were collected one week after the completion of distraction, while tibial samples from the SBD models were collected three weeks post‐surgery. Fresh callus was immersed in ice‐cold RIPA lysis buffer (R0010, Solarbio) containing a protease inhibitor (P6730, Solarbio) and placed in a tissue homogenizer to extract the total tissue protein samples. Similarly, after mechanical TS stimulation or exosome therapy, both BMECs and collected exosomes were extracted on ice for cellular protein samples. The protein concentration of the samples was determined using a BCA assay kit (ZJ102, Epizyme). Equal amounts of protein (20 µg) were separated by 10% (w/v) SDS‐PAGE electrophoresis and transferred onto polyvinylidene difluoride (PVDF) membranes (Millipore, USA). After blocking with a Protein Free Rapid Blocking Buffer (PS108P, Epizyme), the PVDF membranes were incubated overnight with primary antibodies at 4 °C. Then, the membranes were incubated at room temperature with an HRP‐conjugated secondary antibody for 2 h. Protein bands were visualized using a Light Chemiluminescence Kit (SQ202, Epizyme). The intensity of protein bands was semi‐quantitatively analyzed using the ImageJ software (National Institutes of Health, Germany). The antibodies utilized in this study included anti‐YAP (1:1000; A19134, ABclonal), anti‐TAZ (1:1000; A8202, ABclonal), anti‐Notch1 (1:1000; A7636, ABclonal), anti‐Dll4 (1:1000; A12943, ABclonal), anti‐GAPDH (1:5000; AC001, ABclonal), exosome panel (ab275018, Abcam), HRP‐conjugated goat anti‐rabbit antibody (1:10 000; 111‐035‐003, Jackson ImmunoResearch), and light chain specific HRP‐conjugated mouse anti‐rabbit antibody (1:10 000; 211‐032‐171, Jackson ImmunoResearch).

### Co‐Immunoprecipitation (Co‐IP)

An Immunoprecipitation Kit with Protein A+G Magnetic Beads (P2179M, Beyotime) was employed to perform Co‐IP analysis on BMECs following mechanical TS stimulation. Briefly, BMECs were treated with a Lysis Buffer containing a Protease Inhibitor Cocktail on ice after mechanical TS stimulation. The lysates were then centrifuged at 12 000 g for 5 min at 4 °C, and the supernatant was collected. Subsequently, the samples were incubated overnight at 4 °C with specific antibodies or IgG bound to Protein A+G Magnetic Beads. The required antibodies for immunoprecipitation included anti‐RBPJ (1:20; ab25949, abcam), anti‐TEAD1 (1:20; ab133533, abcam), and anti‐TAZ (1:30; ab307148, abcam). Afterward, the samples were placed on a Magnetic Separation Rack for 10 seconds, and the supernatant was removed. SDS‐PAGE Sample Loading Buffer was added to the samples and incubated at 95 °C for 5 min. The samples were then placed on the Magnetic Separation Rack for 10 seconds, and the resulting supernatant was used for western blot analysis as described above.

### Chromatin Immunoprecipitation (ChIP) Assay

After mechanical TS stimulation, BMECs were incubated at 37 °C for 10 min with 1% formaldehyde to crosslink proteins and DNA. Subsequently, the cells were collected at 4 °C and subjected to a ChIP Assay Kit (P2078, Beyotime). Briefly, the cells were washed three times with a pre‐chilled PBS solution containing PMSF (1 mM) and then incubated on ice for 10 min with SDS Lysis Buffer containing 1 mM PMSF to ensure complete cell lysis. Next, the genomic DNA was fragmented into 400–800 bp fragments by sonication at 25% power for five cycles of 10 seconds each, while keeping the samples on ice. After centrifugation at 12 000 g for 5 min at 4 °C, the supernatant was collected. Each 0.2 mL sample was mixed with 1.8 mL ChIP Dilution Buffer containing 1 mM PMSF. Subsequently, the sample was incubated overnight at 4 °C with primary antibodies or IgG. Then, 60 µL of Protein A+G Agarose/Salmon Sperm DNA was added and incubated at 4 °C for one hour for immunoprecipitation. After five washes with Wash Buffer, the obtained sample was incubated in Elution buffer for 5 min, followed by centrifugation at 1000 g for 1 min. The resulting supernatant (500 µL) was mixed with 20 µL of 5 M NaCl and heated at 65 °C for 4 h to reverse the cross–linking between proteins and genomic DNA. The obtained sample was purified using a DNA Purification Kit (D0033, Beyotime) and used for qPCR analysis. The Ct value of the IgG control group was compared to the IP group to calculate the fold enrichment. The antibodies required in this study included anti‐YAP (1:50; #14074, CST) and anti‐Notch1 (1:100; #3608, CST). The primer sequences are listed in Table S1 (Supporting Information).

### Dual‐Luciferase Reporter Assay

Following the construction of human Notch1, Dll4, YAP, and TAZ promoter fragments containing wildtype (prom‐WT) or mutant (prom‐MUT) predicted binding sites, these fragments were inserted into the pGL3‐Basic firefly luciferase (fLUC) reporter vector. Simultaneously, pcDNA3.1 was utilized to construct overexpression vectors for YAP and NICD, as well as control vectors (NC). Subsequently, BMECs were overexpressed with YAP and co‐transfected with Notch1‐prom‐WT, Notch1‐prom‐MUT, Dll4‐prom‐WT, or Dll4‐prom‐MUT using Lipofectamine 2000 reagent (11668019, Invitrogen). Cells transfected with the NC vector served as the control group. Similarly, in another series of experiments, BMECs were overexpressed with NICD and co‐transfected with YAP‐prom‐WT, YAP‐prom‐MUT, TAZ‐prom‐WT, or TAZ‐prom‐MUT. Additionally, the pRL‐TK renilla luciferase (rLUC) control construct was co‐transfected as an internal control plasmid into each group of BMECs. All sequences and vectors used were synthesized and constructed by HANBIO (China). After 48 h of transfection, a Dual‐Luciferase Reporter Assay Kit (HB‐DLR‐100, HANBIO) was employed to measure the ratio of the two fluorescence signals in each group, following the manufacturer's instructions. Briefly, cells from each group were washed with PBS and lysed at room temperature for 15 min using 1× Lysis Buffer. The lysates were then centrifuged at 12 000 rpm at 4 °C for 1 min, and the supernatant was collected. 20 µL of the sample was mixed with 100 µL of Luciferase Reaction Reagent, and the fLUC fluorescence signal was measured after thorough mixing. Subsequently, 100 µL of Luciferase Reaction Reagent II was added, and mixed well, and the rLUC fluorescence signal was measured.

### Exosome Separation, Characterization, and Fluorescence Labeling

After the mechanical TS stimulation treatment, a Serum‐free Media (UR51102, Umibio) was replaced to continue the culture of BMECs for 48 h. Subsequently, exosomes were collected using density gradient centrifugation. In brief, the culture medium was collected and centrifuged at 300 g for 10 min at 4 °C to pellet cell debris. The resulting supernatant was sequentially centrifuged at 2000 g for 10 min, 10 000 g for 30 min, and 100 000 g for 90 min at 4 °C. The pellet obtained from the final centrifugation was resuspended in PBS for further analysis and treatment. Transmission electron microscopy (TEM; HITACHI, Japan) was used to observe the morphology of the exosomes, while the size distribution of the exosomes was determined using a Nanoparticle Tracking Analysis (NTA; Malvern, UK) system. The expression of characteristic markers and proteins in the exosomes was determined by western blot analysis as described above.

To label exosomes with fluorescence, the PKH67 probe (40781ES20, Yeasen) was introduced into the exosome solution, achieving a final concentration of 100 µM. The mixture was then incubated at 4 °C for 30 min. Following this, the fluorescently labeled exosomes were isolated through density gradient centrifugation to remove redundant probes. To evaluate the uptake of exosomes by BMECs as the target cells, the PKH67‐labeled exosomes (50 µg mL^−1^) were added to the culture medium and co‐incubated for a duration of 30 min. Subsequently, F‐actin and cell nuclei were stained using the previously described methods with TRITC‐conjugated phalloidin and DAPI, respectively, and DMi8 fluorescence microscopy (Leica) was employed to observe the cells. In order to determine the distribution of exosomes in vivo, the retention of PKH67‐labeled exosomes within the region of bone defect was assessed using the IVIS Spectrum (PerkinElmer, USA) in vivo imaging system one week after the immediate stereotactic injection of fluorescently labeled exosomes following the surgical procedure.

### Statistical Analysis

The data were expressed as mean ± standard deviation. Statistical analysis was performed using Student's *t*‐test for comparisons between two groups, or one‐way analysis of variance (ANOVA) followed by Tukey's *post‐hoc*‐test for comparisons among multiple groups, utilizing GraphPad Prism 8 software (GraphPad Software, USA). A two‐tailed *p*‐value of <0.05 was considered statistically significant.

## Conflict of Interest

The authors declare no conflict of interest.

## Supporting information

Supporting Information

## Data Availability

The data that support the findings of this study are available from the corresponding author upon reasonable request.
