# Supplementary material for: Tensile Stress‐Activated and Exosome‐Transferred YAP/TAZ‐Notch Circuit Specifies Type H Endothelial Cell for Segmental Bone Regeneration
Source: Adv Sci (Weinh). 2024 Jan 15;11(12):2309133. doi: 10.1002/advs.202309133 (PMC10966515; doi:10.1002/advs.202309133)
Supplement: Supplementary file 1 — Supporting Information [file ADVS-11-2309133-s001.pdf]

ADVANCED SCIENCE
Open Access

Supporting Information

for *Adv. Sci.*, DOI 10.1002/advs.202309133

Tensile Stress-Activated and Exosome-Transferred YAP/TAZ-Notch Circuit Specifies Type H Endothelial Cell for Segmental Bone Regeneration

Feng Wang, Shanyu Li, Lingchi Kong, Kai Feng, Rongtai Zuo, Hanzhe Zhang, Yifan Yu, Kunqi Zhang, Yuting Cao, Yimin Chai, Qinglin Kang and Jia Xu**

Table S1. Primer sequences for quantitative real-time polymerase chain reaction

Gene	Forward (5'-3')	Reverse (5'-3')
YAP	CAGGAGCCCTGACTCCACAG	TTGCCATCTCCCAACCTGCT
TAZ	GCTGGCTCTGATGGCTCTTA	AGACCTGTGACTAATGCTGCTG
Notch1	GGACCAGATTGGGGAGTT	CACACTCGTCCACATCGT
Dll4	ATTGCCAACAGCCTATCT	CCATCCTCCTGGTCCTTA
HES1	TCAACACGACACCGGATAAAC	GCCGCGAGCTATCTTTCTTCA
Acta2	AAAAGACAGCTACGTGGGTGA	GCCATGTTCTATCGGGTACTTC
Cnn1	CTGTCAGCCGAGGTTAAGAAC	GAGGCCGTCCATGAAGTTGTT
GAPDH	CTGGGCTACACTGAGCACC	AAGTGGTCGTTGAGGGCAATG
Notch1 promotor	TCGATCCTCTGGACGCCTAA	GCCTGGGACTACTTCTCGTT
Dll4 promotor	TAGACCCTTAGCCCCTAGCC	ACCTAGTGTTCCAAAGCAGGT
YAP promotor	ACTTGTCCAAAACCTCACAGC	TGAACCCCCAGTTTCCTGTC
TAZ promotor	AGGCAGAGAGGGTTCAGGAT	TCAGGACTCAAAGGGGCTTG

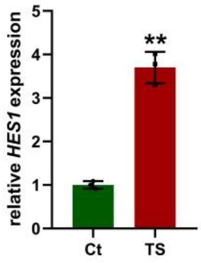

Figure S1 The gene expression of *HES1* in tensile stress (TS)-stimulated bone marrow

endothelial cells. $^{**}P < 0.01$.

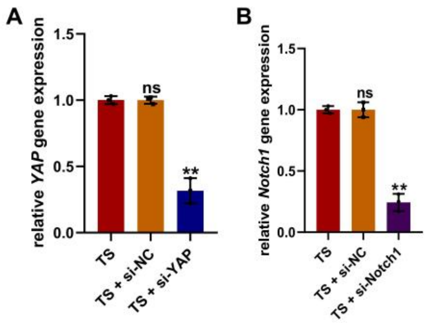

Figure S2 Verification of knockdown efficiency. A-B. The gene expression of *YAP* (A) and *Notch1* (B) in tensile stress (TS)-stimulated bone marrow endothelial cells upon knockdown of *YAP* or *Notch1* by siRNAs, respectively. $^{ns}P > 0.05$, $^{**}P < 0.01$.

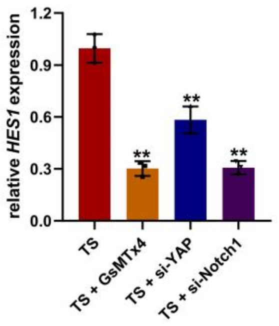

Figure S3 The gene expression of *HES1* after GsMTx4 treatment or knockdown of *YAP* or *Notch1* by siRNAs in tensile stress (TS)-stimulated bone marrow endothelial cells. $^{**}P < 0.01$.

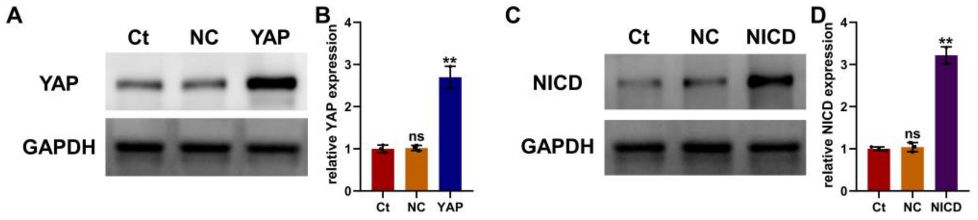

Figure S4 Verification of overexpression efficiency. A-B. Western blot images (A) and quantitative analysis (B) revealing the expression of YAP in transfected bone marrow endothelial cells (BMECs). **C-D.** Western blot images (C) and quantitative analysis (D) revealing the expression of NICD in transfected BMECs. $^{ns}P > 0.05$, $^{**}P < 0.01$.

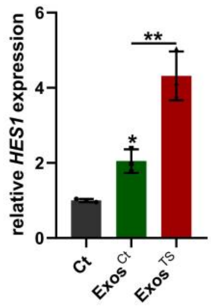

Figure S5 The gene expression of *HES1* in exosomes-treated bone marrow endothelial cells. $^{*}P < 0.05$, $^{**}P < 0.01$.

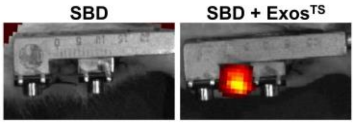

Figure S6 *In vivo* imaging system showing the retention of PKH67-labeled exosomes within the region of bone defect one week after the stereotactic injection.
